# Life without tRNA^Arg^–adenosine deaminase TadA: evolutionary consequences of decoding the four CGN codons as arginine in Mycoplasmas and other Mollicutes

**DOI:** 10.1093/nar/gkt356

**Published:** 2013-05-08

**Authors:** Shin-ichi Yokobori, Aya Kitamura, Henri Grosjean, Yoshitaka Bessho

**Affiliations:** ^1^Laboratory of Extremophiles, Department of Applied Life Sciences, School of Life Sciences, Tokyo University of Pharmacy and Life Sciences, 1432-1 Horinouchi, Hachioji, Tokyo 192-0392, Japan, ^2^RIKEN SPring-8 Center, Harima Institute, 1-1-1 Kouto, Sayo, Hyogo 679-5148, Japan and ^3^Centre de Génétique Moléculaire, UPR 3404, CNRS, Associée à l'Université Paris-Sud 11, FRC 3115, 1 Avenue de la Terrasse, 91190 Gif-sur-Yvette, France

## Abstract

In most bacteria, two tRNAs decode the four arginine CGN codons. One tRNA harboring a wobble inosine (tRNA^Arg^_I__CG_) reads the CGU, CGC and CGA codons, whereas a second tRNA harboring a wobble cytidine (tRNA^Arg^_C__CG_) reads the remaining CGG codon. The reduced genomes of Mycoplasmas and other Mollicutes lack the gene encoding tRNA^Arg^_C__CG_. This raises the question of how these organisms decode CGG codons. Examination of 36 Mollicute genomes for genes encoding tRNA^Arg^ and the TadA enzyme, responsible for wobble inosine formation, suggested an evolutionary scenario where *tadA* gene mutations first occurred. This allowed the temporary accumulation of non-deaminated tRNA^Arg^_A__CG_, capable of reading all CGN codons. This hypothesis was verified in *Mycoplasma capricolum*, which contains a small fraction of tRNA^Arg^_A__CG_ with a non-deaminated wobble adenosine. Subsets of Mollicutes continued to evolve by losing both the mutated tRNA^Arg^_C__CG_ and *tadA*, and then acquired a new tRNA^Arg^_U__CG._ This permitted further tRNA^Arg^_A__CG_ mutations with tRNA^Arg^_G__CG_ or its disappearance, leaving a single tRNA^Arg^_U__CG_ to decode the four CGN codons. The key point of our model is that the A-to-I deamination activity had to be controlled before the loss of the *tadA* gene, allowing the stepwise evolution of Mollicutes toward an alternative decoding strategy.

## INTRODUCTION

The genetic code is composed of 16 families of decoding boxes, each including four codons with the same first two nucleotides. Depending on the amino acid, these synonymous codons are read by one, two or at most three isoacceptor tRNA species harboring distinct anticodons. Therefore, fewer than 61 isoacceptor species (usually between 22 to a maximum of 46) are used to decode the 61 sense codons in mRNAs. These cellular tRNA repertoires are primarily responsible for the efficiency and accuracy of mRNA translation. The tRNA repertoires vary greatly from one organism and organelle to another, with most of the variability being found in the type of nucleotide present at the first ‘so-called’ wobble position of the anticodon (position 34), which is often post-transcriptionally modified. By interacting with the third base of the codon, this frequently modified nucleotide-34 plays an essential role in determining the preferred codons to be read by the mature and functional tRNA (1–5).

**Figure 1.**
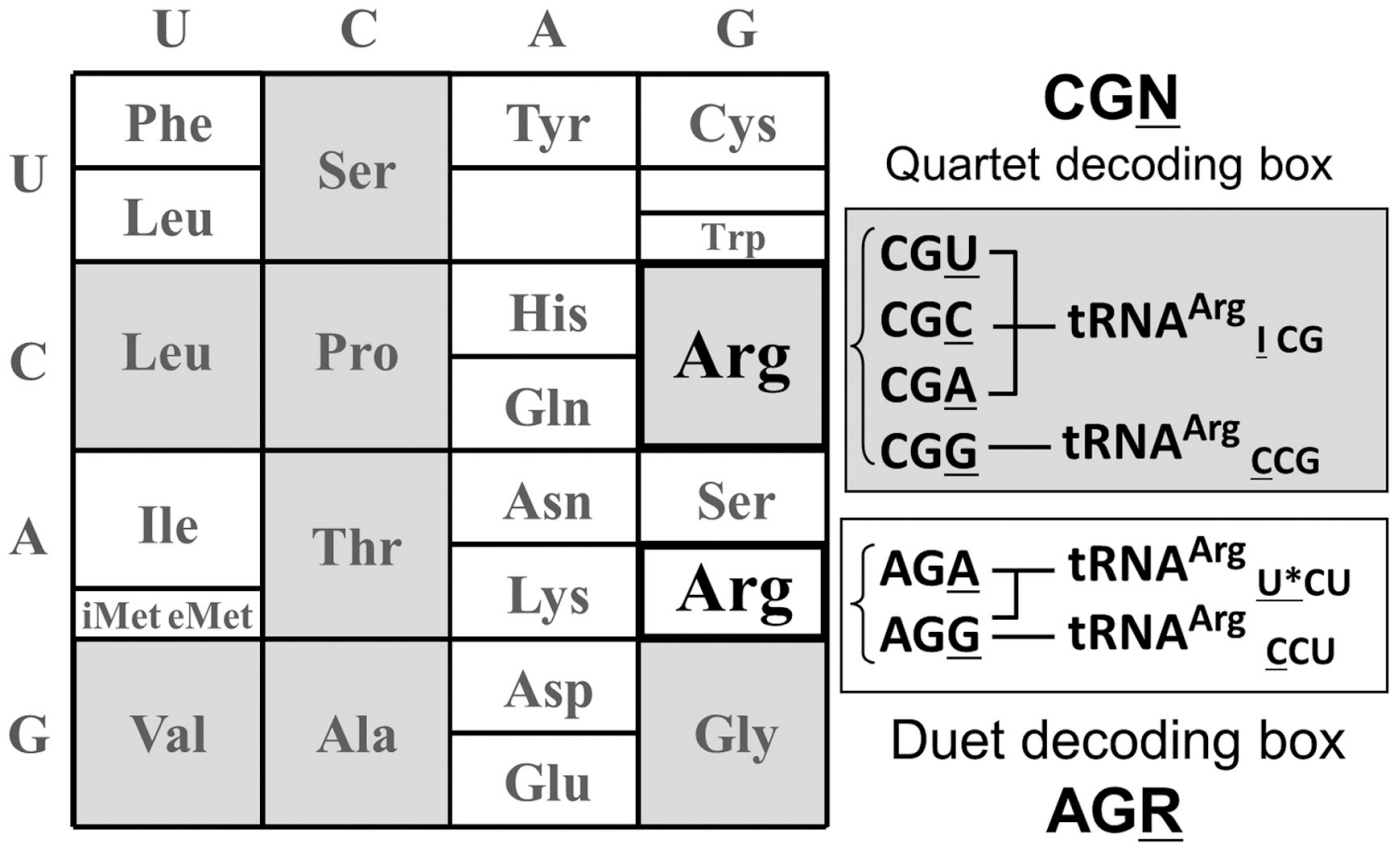
Quartet and duet decoding boxes of the bacterial genetic code, for decoding the 20 amino acids. In the case of arginine, the bacterial tRNA^Arg^ set usually involved in decoding Arg codons is also indicated with the respective anticodons.

Transfer RNAs harboring an unmodified wobble adenosine-34 are rare; thus, they are not frequently used during translation. The reason is that during tRNA maturation, the encoded wobble A_34_ in the anticodon of the precursor tRNAs is generally enzymatically deaminated to inosine (6-deaminated adenosine–hypoxanthine base) by specific tRNA:A_34_ deaminases. The resulting I_34_-containing tRNA was predicted to base pair with a C-ending codon in the Watson–Crick mode and with U- and A-ending codons in a slightly different ‘wobble’ conformation (6), whereas the binding with a G-ending codon was forbidden, as reviewed previously (2,7). However, among the three codons read by I_34_-containing tRNA, the A-ending codon was expected to be difficult to translate, and this proposal was verified with *Escherichia coli* tRNA^Arg^_I__CG_, using an *in vitro* translation system (8). Confirmation of this wobble hypothesis, with both bases in the anti-conformation as initially predicted by Francis Crick, was finally obtained from the crystal structure of the 30S ribosomal subunit, with the anticodon stem loop derived from *E. coli* tRNA^Arg^_I__CG_ bound to the CGA codon in an mRNA fragment (9). Therefore, once a cell has evolved and begun using I_34_-containing tRNA, the fourth remaining codon ending with G, in the corresponding four synonymous codons of the family box, has to be read by a second tRNA isoacceptor harboring a C_34_-containing anticodon (Figure 1). Although this is the usual decoding strategy observed in many living cells (10,11), a few remarkable exceptions exist.

For example, in fungi and animals, all cytoplasmic tRNAs harboring a wobble A_34_ and a purine-35 (R_35_) in the middle of the anticodon, as well as A_34_ in the cytoplasmic tRNA^Arg^_I__CG_, have their wobble base deaminated to inosine-34 by the Tad2/Tad3 heterodimeric enzyme during tRNA maturation (12–14). These I_34_R_35_-containing tRNAs are found in the decoding family boxes using three or four synonymous codons (Leu, Ile, Val, Ser, Pro, Thr and Ala) (11,15). However, in *Arabidopsis thaliana* and other land plants, the same cytoplasmic Tad2/Tad3 deaminase does not deaminate the wobble A_34_ of cytoplasmic tRNA^Arg^_A__CG_, but only those of the other A_34_R_35_-containing tRNAs (16). This raises the question of how the Arg-CGN codons in plant cytoplasmic mRNAs are translated into arginine. Only the chloroplastic tRNA^Arg^_I__CG_ in *A. thaliana* (and probably in all land plants) contains a deaminated A_34_, and its formation is catalyzed by the nuclear encoded chloroplastic TadA, a deaminase that is similar to the bacterial ortholog (17,18).

In contrast to cytoplasmic tRNA of eukaryotes, but similar to plant chloroplasts, inosine-34 in bacterial tRNA is found exclusively in tRNA^Arg^_I__CG_, belonging to the CGN decoding box. Here, the wobble A_34_ is deaminated by a homodimeric tRNA:A_34_-deaminase (TadA) that is specific for only A_34_-containing tRNA^Arg^_A__CG_ (19). No other bacterial tRNAs harboring a wobble A_34_, either naturally occurring or experimentally generated by mutation, are deaminated by TadA. This property facilitated the examination of the decoding properties of A_34_-containing tRNAs other than tRNA^Arg^_A__CG_. Using a mutant tRNA^Pro^_A__GG_ of *Salmonella typhimurium*, in which the naturally occurring wobble G_34_ was mutated to A_34_, Björk and co-workers (20) demonstrated that the C-ending proline codon was read *in vivo* almost as efficiently as the wild-type G_34_-containing tRNA^Pro^_G__GG_. Likewise, a mutant of *E. coli* tRNA^Gly^_C__CC_*,* in which the naturally occurring wobble C_34_ was changed to A_34_ by site-directed mutagenesis, read all four GGN glycine codons, although the A-ending Gly-GGA codon was decoded with the lowest efficiency (21). Osawa and co-workers (22) experimentally proved *in vitro* that the naturally occurring A_34_-containing tRNA^Thr^_A__GU_ from the bacterium *Mycoplasma capricolum* translates all four threonine ACN codons, and only the Thr-ACA codon showed greatly reduced efficiency. Notably, *M. capricolum* has evolved a second tRNA^Thr^_U__GU_ harboring an unmodified wobble U_34_ for reading the ACA codon without wobbling (23); therefore, it has naturally compensated for the difficulty of reading the Thr-ACA codon by A_34_-containing tRNA^Thr^_A__GU_.

As for the mitochondria of the fungus *Saccharomyces cerevisiae* and the nematode *Ascaris suum*, the *tadA* genes are missing in their nuclear genomes, and consequently, their encoded mitochondrial tRNA^Arg^_A__CG_ harbors an unmodified wobble A_34_ (24,25). As no other mitochondrial tRNA^Arg^ belonging to the same CGN arginine box exists, it was concluded that this unique tRNA^Arg^_A__CG_ must decode all four synonymous CGN codons. However, no experiments have been performed to verify this hypothesis.

*Escherichia coli* TadA and cytoplasmic *S. cerevisiae* Tad2/Tad3 are essential enzymes, and the deletions of the corresponding genes are lethal (13,19). Together, these examples demonstrated that, at variance with the information reported in all textbooks, the essential inosine at the first anticodon position does not ‘extend’ the decoding capability of an A_34_-containing tRNA. On the contrary, it ‘restricts’ the precursor tRNA harboring an unmodified wobble A_34_ to read only three of the four potential synonymous codons, excluding only the synonymous codon ending with G. This remaining synonymous G-ending codon of the same decoding box has to be decoded by a C_34_-containing tRNA. However, as aforementioned, although I_34_:A_3_ wobble pairing is possible (9), in practice it is inefficient (8), and cells usually limit the usage of codons involving I_34_:A_3_ base pairing during translation (26–28).

In this report, we identified the tRNA^Arg^ set in the 36 fully sequenced genomes of Mollicutes currently available. This repertoire was then correlated with the presence or absence of a gene encoding a TadA deaminase in the Mollicute genome. This genomic analysis revealed that Mollicutes are evolving by setting up alternative, and probably more efficient, arginine decoding systems able to read all four CGN codons, thus bypassing the requirement for the usually essential bacterial *tadA* gene.

## MATERIALS AND METHODS

### Data processing

All bacterial genomes analyzed were obtained from Genbank. They are listed in Supplementary Table S1. The genes encoding the TadA (tRNA-specific adenosine deaminase) and CDA (cytidine deaminase) protein sequences from the different Mycoplasmas analyzed were obtained from Genbank via BLASTP at NCBI, using TadA of *Bacillus subtilis* subsp. *subtilis* str. 168 (NP_387899.1) as the query sequence under the default conditions. The sequences of a few additional bacterial TadA proteins were obtained from published articles (Table 1). The tRNA^Arg^ genes with the anticodons ACG, GCG, TCG, CCG (belonging to the quartet decoding arginine box) and TCT or CCT (belonging to the duet decoding arginine box) were retrieved and listed in one file (Supplementary Figure S1). The two available tRNA^Arg^ sequences (including indications of their modified nucleotides) from Mycoplasmas, and the sequences of 35 tRNAs specific for other amino acids, were obtained from the tRNADB-CE databank (http://trna.nagahama-i-bio.ac.jp) (32) and tRNAdb (http://trnadb.bioinf.uni-leipzig.de) (15). Two additional sequences of tRNA^Arg^ from *Acholeplasma laidlawii* (anticodon branch only) were obtained from a published report (33). The numbers of occurrences of each Arg-codon in mRNA were counted directly from each genome sequence obtained from Genbank. The phylogenies of Mollicutes were obtained from the MolliGen 3.0 database (http://cbib1.cbib.u-bordeaux2.fr/molligen3b/SPECIES/phylo.php) (29).

**Table 1.** Comparative usage of Arg codons, number of tRNA^Arg^ genes and occurrence of the *tadA* gene in 10 bacterial and 36 parasitic Mollicute genomes

Number	Species	Group	Number of Arg codons in ORFs						Anticodon and number of tRNA genes						Gene
			*CGU*	*CGC*	*CGA*	*CGG*	*AGA*	*AGG*	*ACG*	*GCG*	*TCG*	*CCG*	*TCT*	*CCT*	*tadA*
1a	*E. coli* str. K-12 substr. MG1655	Outer	28 485	29 996	4871	7432	2845	1651	**4**			**1**	**1**	**1**	**1**
1b	*Nitrosomonas europaea* ATCC 19718	Outer	13 425	14 553	4584	10 153	5082	3473	**1**			**1**	**1**	**1**	**1**
1c	*A. aeolicus* VF5	Outer	727	601	268	367	9229	12588	**1**			**1**	**1**	**1**	**1**
1d	*Streptomyces avermitilis* MA 4680	Outer	19 076	93 823	7656	74019	2208	9827	**1**			**1**	**1**	**1**	**1**
1e	*Synechococcus elongatus* PCC 6301	Outer	8173	24 198	8010	787	24 448	1135	**1**			**1**	**1**	**1**	**1**
1f	*S. aureus* subsp. *aureus* Mu50	Outer	10 775	2603	3956	388	9321	1202	**2**			**1**	**1**		**1**
1g	*Bacillus cereus* ATCC 14579	Outer	20 003	6523	7745	1911	13 891	3604	**4**			**1**	**1**	**1**	**1**
1h	*B. subtilis* subsp*. subtilis* str. 168	Outer	9150	10 389	4957	7839	13 194	4700	**4**			**1**	**1**	**1**	**1**
1i	*Listeria monocytogenes* EGDe	Outer	10 836	6301	5099	2578	5899	1102	**2**			**1**	**1**	**1**	**1**
1j	*Oenococcus oeni* PSU1	Outer	5934	2698	2965	2152	3951	1353	**1**			**1**	**1**	**1**	**1**

2	*A. laidlawii* PG-8A	IV	4075	872	747	**61**	8639	670	**1**				**1**		**1**
3	*Aster yellows witches'-broom phytoplasma* AYWB	IV	1183	710	305	**42**	2109	222	**1**				**1**		**1**
4	*Candidatus Phytoplasma australiense*	IV	1332	804	505	**77**	2484	358	**1**				**1**		**1**
5	*Candidatus Phytoplasma mali*	IV	1047	122	350	**33**	1972	168	**1**				**1**		**1**
6	*Onion yellows phytoplasma* OY-M	IV	1455	843	379	**58**	2457	237	**1**				**1**		**1**

7	*Mesoplasma florum* L1	I	996	66	127	**2**	5444	190	**1**				**1**		**1**
8	*M. capricolum* subsp. *capricolum* ATCC 27343	I	904	100	153	**6**	6115	184	**1**				**1**		**1**
9	*Mycoplasma leachii* PG50	I	931	107	147	**5**	6154	175	**1**				**1**		**1**
10	*M. mycoides subsp. mycoides* SC str. PG1	I	1061	95	167	**10**	7324	272	**1**				**1**		**1**
11	*M. mycoides subsp. capri* LC str. 95010	I	1048	107	157	**9**	7275	252	**1**				**1**		**1**

12	*Mycoplasma agalactiae*	III	1349	258	153	56	6250	711	**1**				**1**	**1**	
13	*M. agalactiae* PG2	III	1186	255	163	57	5296	653	**1**				**1**	**1**	
14	*Mycoplasma arthritidis* 158L3-1	III	1975	806	633	262	3233	327	**1**				**1**	**1**	
15	*Mycoplasma bovis* PG45	III	1256	289	191	65	6129	758	**1**				**1**	**1**	
16	*Mycoplasma conjunctivae* HRC/581	III	1786	718	728	175	3500	365	**1**				**1**		
17	*M. crocodyli* MP145	III	910	94	122	23	5564	337	**2**				**1**	**1**	
18	*Mycoplasma hominis* ATCC 23114	III	899	181	136	46	3818	413	**1**				**1**	**1**	
19	*Mycoplasma hyopneumoniae* 232	III	1485	938	1211	721	2858	745	**1**				**1**		
20	*M. hyopneumoniae* 7448	III	1463	938	1210	672	2852	719	**1**				**1**		
21	*M. hyopneumoniae* J	III	1460	933	1196	665	2881	710	**1**				**1**		
22	*Mycoplasma hyorhinis* HUB-1	III	913	125	340	41	4881	297	**1**				**1**		
23	*M. mobile* 163K	III	618	77	171	26	5441	411	**1**				**1**		
24	*Mycoplasma synoviae* 53	III	986	136	96	60	4811	284	**1**				**1**		

25	*M. fermentans* JER	III	2030	258	314	63	5371	236	**1**		**1**		**1**	**1**	
26	*M. fermentans* M64	III	2164	303	335	81	6439	315	**1**		**1**		**1**	**1**	
27	*Mycoplasma penetrans* HF-2	II	467	15	52	26	8579	492	**1**		**1**		**1**		
28	*Ureaplasma parvum serovar* 3 str. ATCC 27815	II	3098	447	946	122	1571	122	**1**		**1**		**1**		
29	*Ureaplasma parvum serovar* 3 str. ATCC 700970	II	3087	450	946	122	1592	127	**1**		**1**		**1**		
30	*Ureaplasma urealyticum serovar* 10 str. ATCC 33699	II	3671	369	1044	90	1652	77	**1**		**1**		**1**		

31	*M. gallisepticum* str. R(low)	II	2031	616	925	498	4846	446		**2**	**1**		**1**		
32	*Mycoplasma genitalium* G37	II	1226	540	239	185	2439	812		**1**	**1**		**1**	**1**	
33	*Mycoplasma pneumoniae* M129	II	2340	2579	599	1200	968	679		**1**	**1**		**1**	**1**	
34	*M. pulmonis* UAB CTIP	III	329	205	538	277	7228	2289		**1**	**1**		**1**		
35	*Mycoplasma suis* KI3806	II	109	55	306	49	6337	717		**1**	**1**		**1**		
36	*M. suis* str. Illinois	II	123	71	355	61	6785	788		**1**	**1**		**1**		

37	*M. haemofelis* str. Langford 1	II	887	282	810	324	5889	3495			**1**		**1**		

The frequencies of arginine codons in protein-encoding ORFs in each genome were obtained from Genbank. The information about the presence or absence of a given tRNA^Arg^ gene (the number corresponds to the number of genes encoding a tRNA with a given anticodon), as well as that about the *tadA* gene (always one when present), was obtained from the NCBI genome database, using BLASTN and BLASTP searches, respectively. The third base of the codon and the first wobble base of the anticodon are underlined. The accession numbers of the species, the subfamilies to which they belong and their hosts (in the cases of parasitic Mollicutes), their genome sizes, G + C% and references are provided in Supplementary Table S1. Species 2–6 correspond to Mollicutes of Group IV (Phytoplasmas), species 7–11 correspond to Mollicutes of Group I (Spiroplasmas), species 12–26 + 34 correspond to Mollicutes of Group III (Hominis) and finally species 27–33 and 35–37 correspond to Mollicutes of Group II (Pneumoniae). Descriptions of the different classes of Mollicutes are available (29–31). The CGG codon usages of Mollicute Groups IV (Phytoplasmas) and I (Spiroplasmas) are highlighted in bold letters.

### Alignment of TadA amino acid sequences

As the amino acid sequences of TadA and CDA are difficult to distinguish by a simple BLAST homology search, we first aligned TadA and CDA. After identification of the genes encoding TadA, we created a second alignment of only the TadAs from the species listed in Supplementary Table S1, using Clustal X 2.0.12 (34) under the default conditions. The TadA enzyme catalyzes the deamination of wobble A_34_-containing tRNA, whereas the CDA enzyme catalyzes the deamination of free cytidine to produce uridine. As the TadAs are apparently derived from an ancestral CDA (35), the comparison allowed us to assess the conserved amino acids and to distinguish the ones that are ‘mechanistically’ common to all members of the deaminase superfamily (CDA and TadA) from those that are specific to TadA, such as those composing the tRNA-binding motif.

### cDNA analyses of *M. capricolum* and *B. subtilis* tRNA^Arg^

**Figure 2.**
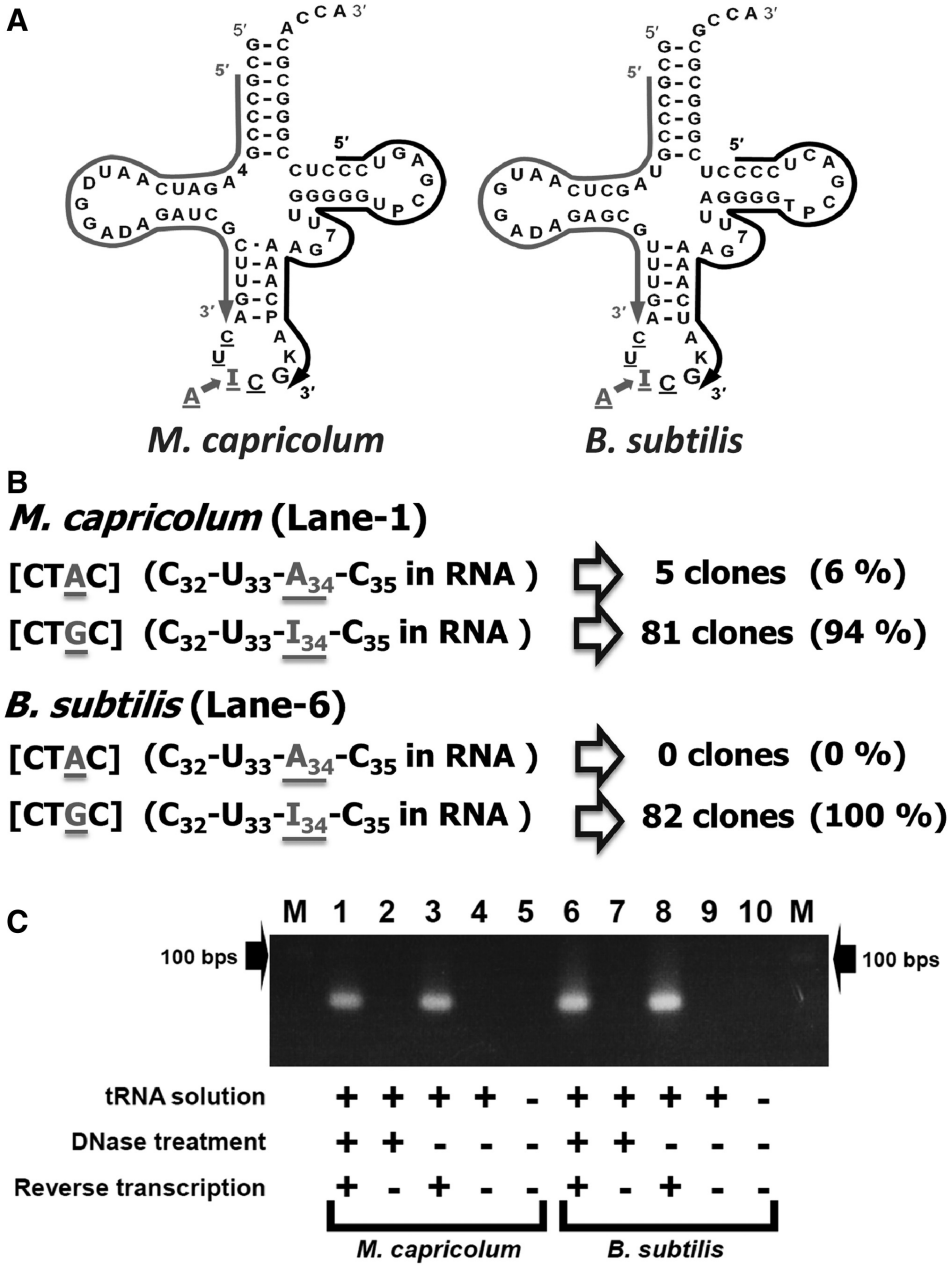
Reverse transcriptase–PCR of tRNA^Arg^_I__CG_ from *M. capricolum* and *B. subtilis*. (**A**) Comparison of the nucleotide sequences of *M. capricolum* (Mca) and *B. subtilis* (Bsu) tRNA^Arg^_I__CG_, obtained from (15). The cloverleaf structures are shown. I, 4, D, K, P, 7 and T represent inosine, 4-thio-uridine, dihydrouridine, 1-methylguanosine, pseudouridine, 7-methylguanosine and 5-methyluridine (ribosylthymine), respectively. Regions of primers for reverse transcription of the first strand (and first primers for PCR) are shown with black arrows. Regions of the second primers for PCR are shown with gray arrows. (**B**) Summary of sequences of cDNA clones for *M. capricolum* and *B. subtilis* tRNA^Arg^_I__CG_. The DNA sequences of the cDNA clones, except for the PCR primer regions, are shown in brackets. The RNA sequences corresponding to the obtained DNA sequences are shown in parentheses. I (inosine) in the RNA sequence corresponds to G in the DNA sequence obtained by reverse transcription. (**C**) Agarose gel electrophoresis of reverse transcriptase–PCR products. Lane M: size marker (100-bp ladder, the position of 100 bp is shown with an arrow). Lanes 1–10: PCR products of various templates. Lane 1: reverse-transcribed McatRNA^Arg^_I__CG_ solution treated with DNase before reverse transcription. Lane 2: total McatRNA solution with DNase treatment. Lane 3: Reverse-transcribed McatRNA^Arg^_I__CG_ solution without DNase treatment before reverse transcription. Lane 4: total McatRNA solution without DNase treatment. Lane 6: reverse-transcribed BsutRNA^Arg^_I__CG_ solution with DNase treatment before reverse transcription. Lane 7: total BsutRNA solution with DNase treatment. Lane 8: reverse-transcribed BsutRNA^Arg^_I__CG_ solution without DNase treatment before reverse transcription. Lane 9: total BsutRNA solution without DNase treatment. Lanes 5 and 10: control (no RNA/DNA).

Bulk tRNA from *B. subtilis* strain 168 (wild-type) was obtained as described previously (36). Bulk tRNA from *M. capricolum* [American Type Culture Collection 27343 (kid)] at the late-log growth phase was obtained by the same procedure. Twenty micrograms of total tRNA from either *M. capricolum* or *B. subtilis* was treated with 4 U of Turbo DNase (Ambion), in the presence of 80 U of RNaseOUT (Invitrogen) for 30 min at 37°C. Following the suppliers’ protocols, the Turbo DNase was removed first, and then reverse transcription for first strand cDNA synthesis was performed, using 0.2 μg of total tRNA and 200 U of SuperScript III reverse transcriptase (Invitrogen). The primers for first strand cDNA synthesis of *M. capricolum* tRNA^Arg^ and *B. subtilis* tRNA^Arg^ were 5′-GGACT-CGAAC-CCCCA-ACCTT-TTGAT-CC-3′ (Mca-1st) and 5′-GGGAG-TCGAA-CCCCT-AACCT-TTTGA-TCC-3′ (Bsu-1st), respectively (black arrows in Figure 2A). In addition to the first strand cDNA synthesis primers, the following primers 5′-GCCCG-TAGAT-CAATT-GGATA-GATCG-CTTGA-3′ (Mca-2nd) and 5′-GCCCG-TAGCT-CAATG-GATAG-AGCGT-TTGA-3′ (Bsu-2nd) were used for further polymerase chain reaction (PCR) amplification of the cDNAs (gray arrows in Figure 2A). Aliquots (2 μl) of the aforementioned reaction mixtures, containing both types of primers, were incubated with 2.5 U of EX Taq DNA polymerase Hot Start version (TAKARA) in a 50 μl reaction solution, using a GeneAmp PCR System 9700 (Applied Biosystems, Life Technologies) thermal cycler. The final concentrations of primers and dNTPs were 400 nM and 200 μM (each), respectively. After pre-heating the PCR solution at 96°C for 4 min, 25 cycles of thermal denaturation/annealing/polymerization steps were performed (10 s at 98°C, 10 s at 50°C and 60 s at 72°C, respectively). The cDNA amplification products were analyzed by 4% agarose (MetaPhor™ Agarose, Lonza Co.) gel electrophoresis in Tris-borate-EDTA (TBE) buffer, using 100-bp size markers (New England Biolabs) to evaluate the lengths of the PCR transcripts. The recovered cDNAs were then cloned, using a TOPO-TA cloning kit for sequencing (Invitrogen). The plasmids were purified with a Montage Plasmid Miniprep_HTS_ 96 kit (Millipore), using a Biomek 2000 (Beckman Coulter). A BigDye Terminator 3.1 kit (Applied Biosystems) was used for sequencing reactions, and a PRISM 3130xl DNA Autosequencer (Applied Biosystems) was used for sequencing. The obtained sequences were analyzed with the Geneious 5.6.5 software (Biomatters).

### Comparison of the 3D structure of *Staphylococcus* TadA and the putative 3D structure of TadA from *M. capricolum*

A homology model of TadA from *M. capricolum* was created, based on its amino acid sequence and the crystal structure of TadA in complex with RNA from *Staphylococcus aureus* (PDB code: 2B3J) (37), using the SwissModel automatic modeling server from Expasy (http://swissmodel.expasy.org/). The hydrogen bonded contacts between TadA and tRNA were calculated by the LIGPLOT programs (38). Structure representations were prepared with the Pymol program (Schrödinger, LLC).

## RESULTS

### Decoding arginine codons in Mollicutes

Table 1 lists the frequencies of codon usage for each of the six arginine codons (4× CGN and 2× AGR, Figure 1), together with the corresponding usage of the tRNA^Arg^ isoacceptors, classified according to their anticodons (NCG and YCU) in 36 Mollicutes. This range of Mollicutes, all with reduced genome sizes (Supplementary Table S1), thoroughly covers the four major clades of the monophylogenetic phylum of this group of bacteria, i.e. Group I for Spiroplasma (items 7–11), Group II for Pneumoniae (items 27–37, except for 34 belonging to Group III), Group III for Hominis (items 34 + 12–26) and Group IV for Phytoplasma and Acholeplasma (items 2–6). For comparison, the situations in a few selected bacterial genomes outside the Mollicute family (items 1a–1j) are also shown. The table includes information about the presence or absence of a gene encoding a homolog of *B. subtilis* TadA (accession No. NP_387899.1), as query sequence. The E-values of the candidate protein sequences in the BLASTP search are >1e-13 (10^−^^13^). No other Mollicute proteins showed E-values >1e-09 (10^−^^9^).

Inspection of Table 1 leads to the following conclusions:

- In contrast to most bacteria, no gene encoding a tRNA^Arg^ harboring the same anticodon is redundant. This trend fits with the gene economization strategy used by Mollicutes, with their small genome sizes. The only exception is for tRNA^Arg^_G__CG_ in *Mycoplasma gallisepticum,* which is encoded by two genes differing by only a single base at position 25 in the D-stem (C_25_ or A_25_), thus creating a mismatch G_10_-A_25_ in one of the two tRNAs (Supplementary Figure S1, and indicated in the Group II- Pneumoniae of Supplementary Figure S2). *Mycoplasma crocodyli* also has two genes encoding tRNA^Arg^_A__CG_ in its genome; however, these have exactly the same sequence (Supplementary Figure S1).
- In contrast to most bacteria, none of the Mollicutes examined carries a gene encoding C_34_-containing tRNA^Arg^_C__CG_ (row 13 in Table 1). This gene was obviously already lost in the genome of the common ancestor of Mollicutes. The lack of this gene is correlated with a drastic reduction, but not the complete elimination, of the CGG codons in mRNA (row 7 in Table 1), which are normally read by the missing tRNA^Arg^_C__CG_, especially in Spiroplasma (Group I, items 7–11) and Phytoplasma (Group IV, items 2–6, indicated in bold in Table 1). An analysis of the ORFs containing the few remaining Arg-CGG codons revealed that they are often used in genes encoding DNA and RNA modification enzymes, with only one codon in each gene, such as in Dam and DNA methylases, TruA, TruB, ThiI (indicated in bold in Supplementary Table S2) and even the tRNA-A_34_ deaminase TadA (indicated in bold and italics in the same Supplementary Table S2). The presence of a problematic Arg-CGG codon at the beginning (second position) of the mRNA corresponding to the *tadA* gene of *Mycoplasma mycoides* (Spiroplasma) is notable, and it suggests that the level of TadA deaminase expression in this organism may depend on the ability of the remaining single tRNA^Arg^ of the Arg-CGN decoding box to read this rare CGG codon.
- All Mollicutes belonging to Groups III (Hominis, items 12–26 and 34) and II (Pneumoniae, items 27–33 and 35–37) lack the *tadA* gene, whereas in all Mollicutes of Groups IV (Phytoplasma, items 2–6) and I (Spiroplasma, items 7–11), the *tadA* gene is still present. The corollary is that A_34_, in the remaining single tRNA^Arg^_A__CG_ of the quartet decoding box, should normally be matured into I_34_ in all Groups I and IV Mollicutes, whereas in Groups II and III, the encoded wobble A_34_ will remain unmodified. Thus, the absence of the tRNA deaminase TadA in the Groups II and III Mollicutes obviously does not affect the viability of these cells, which have also adopted the strategy of preferring the arginine codon usage to mostly AGA of the duet decoding box (Table 1, compare the frequencies of codon usage in row 8 in Mollicutes—items 12–37, with those for bacteria—items 1a–1j). Groups I and IV of the Mollicutes (items 2–11) pose a more difficult problem because the cells have to read the four CGN codons with only a single I_34_-containing tRNA^Arg^_I__CG_, which is normally unable to read CGG. Here, the dramatic reduction in CGG codon usage (indicated in bold in Table 1) and the preference for using the codon AGA of the duet decoding box instead is evident, especially in Spiroplasma (Group I, items 7–11). This AGA arginine codon will be read by the modified U*_34_-containing tRNA^Arg^_U*__CU_ belonging to the duet decoding arginine box (see later in the text).
- All Mollicutes of Group II (Pneumoniae), and *Mycoplasma fermentans* plus *Mycoplasma pulmonis* belonging to Group III-Hominis, have an additional tRNA^Arg^ harboring the anticodon UCG (row 12 in Table 1, items 25–37), thus alleviating the difficulty of reading both codons ending with A and G by A_34_- or I_34_-containing tRNA^Arg^. Moreover, in most Pneumoniae with *M. pulmonis* (items 31–36), the A_34_-containing tRNA^Arg^_A__CG_ is replaced by the G_34_-containing tRNA^Arg^_G__CG_. Together with the U_34_-containing tRNA^Arg^_U__CG_, this allows all four CGN arginine codons to be easily read, in contrast to the Hominis clade (items 12–24), with only a single A_34_-containing tRNA^Arg^_A__CG_. Only *Mycoplasma haemofelis* (Pneumoniae, item 37) remains with a single tRNA^Arg^ harboring the UCG anticodon, with the wobble U_34_ probably kept unmodified to enable the reading of all four CGN codons by ‘superwobbling (four-way wobbling)’ (22,39,40).
- The only tRNA^Arg^ present in all Mollicutes analyzed is tRNA^Arg^_U*__CU_ of the duet decoding Arg-box (Figure 1 and Table 1), where U* stands for 5-carboxymethylaminomethyluridine (cmnm^5^U), as demonstrated in *M. capricolum* tRNA^Arg^_U*__CU_ (41). The modification of U_34_ in this tRNA^Arg^_U*__CU_ is catalyzed by the multi-protein complex MnmE/MnmG present in almost all bacteria, including Mollicutes (42,43). Together with a second C_34_-containing tRNA^Arg^_C__CU_ of the same duet decoding arginine box (only present in a few Mollicutes, Table 1), they translate the frequently used Arg codons AGA and AGG (AGR). From an evolutionary point of view, the existence of a second decoding box for arginine probably greatly facilitated the progressive shift in the decoding strategy within the other arginine decoding box.

### In *M. capricolum*, the wobble A_34_ of a small fraction of tRNA^Arg^_A__CG_ is not deaminated

The nucleotide sequence of the naturally occurring tRNA^Arg^_I__CG_ of *M. capricolum* has been sequenced (41). However, no information was provided about the possibility that a small fraction of this tRNA population was not completely matured, especially at the wobble A_34_ position (Figure 2A). To clarify this point, we sequenced the anticodon region of cDNA^Arg^_I__CG_, obtained after reverse transcription of the naturally occurring tRNA^Arg^_I__CG_ present in the bulk tRNA of *M. capricolum* (Figure 2A). As inosine behaves like G during transcription, we expected to obtain a G at the corresponding position in the cDNA^Arg^. In contrast, if a fraction of the wobble A_34_ in the tRNA sample is not modified into I_34_, then some cDNA^Arg^ clones will now carry A at position 34, and the proportion of ‘A’-clones over ‘G’-clones will provide information about the degree of A_34_-to-I_34_ modification in the original *M. capricolum* tRNA sample. As shown in Figure 2B (upper part), among 86 cDNA clones analyzed, 5 clones (6%) have A at the anticodon first position, and the remaining 81 cDNA clones have G (94%). To confirm this result, several control experiments were performed. First, when the reverse-transcribed tRNA solution was used as the PCR template, only the cDNAs of *M. capricolum* tRNA^Arg^_I__CG_ were amplified (Figure 2C, lanes 1 and 3). Second, in the absence of reverse transcriptase, no cDNA products were PCR amplified (Figure 2C, lanes 2 and 4), confirming the absence of DNA contamination (even without DNase treatment). The results shown in Figure 2B were obtained using the cDNA shown in lane 1 of Figure 2C. The second series of control experiments involved performing the same analysis with bulk tRNA obtained from *B. subtilis* (Figure 2B and C). The tRNA^Arg^_I__CG_ sequence in this bacterium is similar to its *M. capricolum* homolog (Figure 2A) (15). The results from the analysis of 82 clones obtained from the cDNA (lane 6 in Figure 2C) indicated that, in contrast to the bulk tRNA from *M. capricolum*, no clone contained a cDNA^Arg^ with an A at the anticodon position 34, and only G_34_ was detected (100% - Figure 2B), corresponding to the fully matured I_34_ in the original sample of *B. subtilis* tRNA^Arg^_I__CG_. These experiments demonstrated that in naturally occurring *M. capricolum* cells, a minor fraction of tRNA^Arg^ with unmodified wobble A34 (anticodon ACG) does exist and probably functions in translating all Arg-CGN codons (21,22).

### The enzymatic deamination of A_34_ in tRNA^Arg^_A__CG_ in Mollicutes is probably not as efficient as in other bacteria

**Figure 3.**
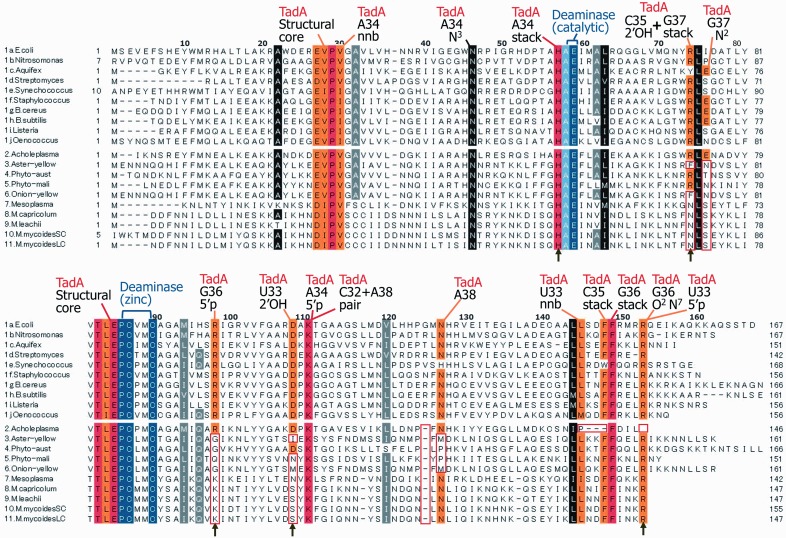
Amino acid sequence alignment of the genes encoding TadA. The TadA amino acid sequences from the species listed in Table 1 were retrieved from Genbank and aligned by Clustal X (34), under the default conditions. The amino acid numbers from *E. coli* are indicated above the alignment. The amino acid numbers from other species are indicated at the beginning and the end of the sections. The TadA-specific conserved amino acids are highlighted with a red or orange background. The conserved amino acids common among TadA and CDA are highlighted with a black or gray background. The conserved deaminase catalytic and zinc-binding sequences are highlighted in blue or light blue. Structurally and functionally important residues of TadA, inferred from the tertiary structures of the *A. aeolicus* and *S. aureus* TadAs (37,44), are indicated above the alignment. The terms ‘nnb’ and ‘stack’ mean non-bonded (hydrophobic) contacts and stacking interactions, respectively. The red boxes in Mollicutes (sequences 2–11) indicate the variations from other bacterial TadAs (sequences 1a–1j). Conserved amino acids involved in tRNA interactions, which are depicted by stick models in Figure 4, are indicated by arrows below the sequences.

**Figure 4.**
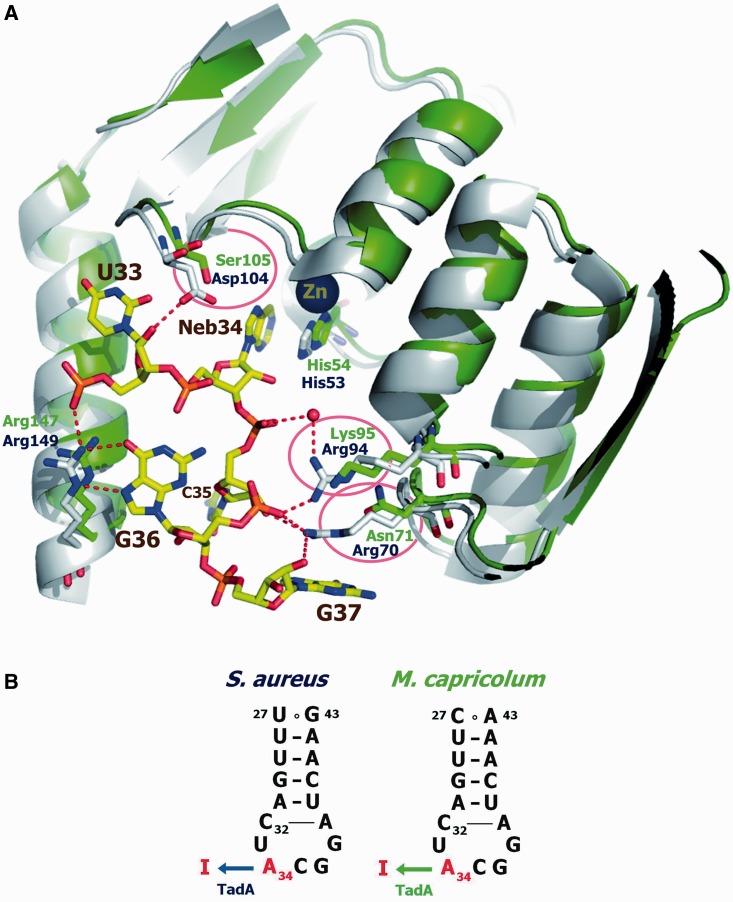
(**A**) Homology model of *M. capricolum* TadA, superposed on *S. aureus* TadA complexed with tRNA^Arg^_A__CG_. Both TadA proteins are represented by ribbon models, colored green for *M. capricolum* and gray for *S. aureus*. The *S. aureus* tRNA is depicted by a stick model. Conserved amino acids involved in tRNA interactions, which are indicated by arrows in Figure 3, are shown in stick models. The amino acids specific to Mycoplasma, indicated in the red boxes in Figure 3, are circled. (**B**) Sequences of the anticodon branches of the tRNA^Arg^_A__CG_ from *S. aureus* and *M. capricolum* (15).

A small fraction of non-deaminated tRNA^Arg^_A__CG_ may also exist in other Mollicutes with genomes encoding *tadA*. This possibility could result from insufficient *tadA* gene expression and/or an abnormally inefficient (degenerate) deaminase. To examine this latter possibility, we compared the amino acid sequences of 10 TadA proteins encoded in the genomes of various bacteria (sequences 1a–1j in Figure 3), with those of 10 TadA proteins of the Mollicutes of Groups I (Spiroplasma) and IV (Phytoplasma), all encoding the *tadA* gene (sequences 2–11 in Figure 3). The list includes the well-characterized TadAs from *E. coli* (sequence 1a) (19,45), *Aquifex aeolicus* (sequence 1c) (44) and *S. aureus* (sequence 1f) (37). The amino acids with identical locations in the sequences are highlighted with black or colored backgrounds, and the systematic sequence deviations among these invariant or semi-invariant amino acids are boxed. The correspondence of these remarkable amino acids within the architecture of the TadA enzyme (indicated with black and colored backgrounds), and of the nucleotide position in tRNA (indicated in black), is depicted at the top of the figure. This information was deduced from the crystal structure of *S. aureus* TadA in complex with a chemically synthesized anticodon stem loop (16mer) bearing nebularine-34 as a substrate, in place of inosine-34 (Figure 4A) (37). For clarity, all other important elements of the anticodon branch in contact with the deaminase are not shown, as they are similar in the tRNA^Arg^_A__CG_ of both *S. aureus* and *M. capricolum* (Figure 4B).

Among the important invariant amino acids to be considered in the A-to-I deaminase TadA, some are also common within the C-to-U deaminase CDA (35), including the AE motif of the deaminase catalytic center, and PCxxC of the zinc-binding motif (Figure 3). In addition, the TadA proteins from Mollicutes (sequences 2–11) share several other identity elements in common with some selected bacterial TadA proteins (red or orange background), i.e. the EVPV and TLE motifs of the TadA-structural core, and several amino acids at conserved positions, such as His57, Lys111 and Phe149 (*E. coli* numbers), which is precisely the region in contact with the tRNA anticodon loop (37). More interesting are the systematic sequence deviations and the absence of certain amino acids (gaps, indicated by dashes) in the TadA sequences of Mollicutes (sequences 2–11, positions in red boxes), as compared with the TadA sequences of other bacteria.

To better visualize the implications of these different amino acids within the active site architecture of the deaminase, the sequence of TadA from *M. capricolum* (item 8 in Figure 3) was superposed on the 3D architecture of TadA from *S. aureus* (item 1f in Figure 3) in complex with a 16 nt mini substrate. As shown in Figure 4A, it is now clear that Asn71 and Lys95 in *M. capricolum* (indicated in green and encircled in red) replaced Arg70 and Arg94 in *S. aureus* (indicated in blue). Therefore, the ribose phosphate backbone of nucleotides G_37_ and G_36_ in the anticodon loop, which H-bond with these amino acids in the case of the *S. aureus* TadA–RNA complex, may not be well fixed, or exist in a slightly different configuration in the case of the putative complex of the same RNA with *M. capricolum* TadA. Moreover, in the vicinity of the essential zinc motif and nebularine-34, and thus within the catalytic center of the deaminase, Ser105 (indicated in green and encircled in red) in *M. capricolum* replaces the important Asp104 in *S. aureus* (indicated in blue), which normally H-bonds with the ribose of U at position 33, adjacent to nucleoside 34 of the anticodon loop. The absence of an interaction with the ribose of U_33_, together with the absence of H-bonding because of the amino acid replacements at positions 70/71 and 94/95 discussed earlier in the text, may affect the dynamics (flexibility/adaptability) of the entire anticodon branch within the active site of the deaminase. Consequently, this may limit the accessibility of the amine target of the wobble A_34_ for deamination, which is catalyzed by the neighboring zinc atom (in the brown background) around His-53/54.

A global inspection of the 3D architecture of *S. aureus* TadA in complex with its RNA mini substrate (37) revealed that the A_31_-U_39_ base pair at the beginning of the anticodon stem does not interact with any amino acids of the deaminase. Only the C_32_-A_38_ pair interacts with Lys106 and Asn123 (Supplementary Figure S3). However, Lys106 (Lys107 in *M. capricolum*) is conserved in all TadA proteins examined (Figure 3), whereas Asn123 (Asn122 in *M. capricolum*) is replaced with different amino acids among the various Mollicutes; therefore, it may not be important for the catalytic function of the deaminase. It is likely that only the mutations in the tadA* gene corresponding to the catalytic core of the deaminase, as discussed earlier in the text, contribute to the modulation of the A_34_-deamination efficiency and ultimately play a role in decoding all four arginine CGN codons.

## DISCUSSION

During protein synthesis, tRNAs bearing the complementary anticodons read mRNA codons. However, because different types of relaxed base pairing are allowed between the often modified ‘wobble’ base at position 34 of the anticodon and the last nucleotide of the codon, some tRNA species can read two, three or even four synonymous codons. Therefore, the number of isoacceptor tRNAs with distinct anticodons needed to read all synonymous codons of a given amino acid is usually lower than the number of codons specifying that particular amino acid in the genetic code. Various organisms apply different rules to adapt their tRNA sets, attesting to the existence of distinct cellular strategies for reading the almost universal genetic code (4). Here, we focused on reading the quartet arginine codons in the quickly evolving Mollicutes with reduced genomes (0.6–1.5 Mb, Supplementary Table S1).

### Reading arginine codons in *M. capricolum*

**Figure 5.**
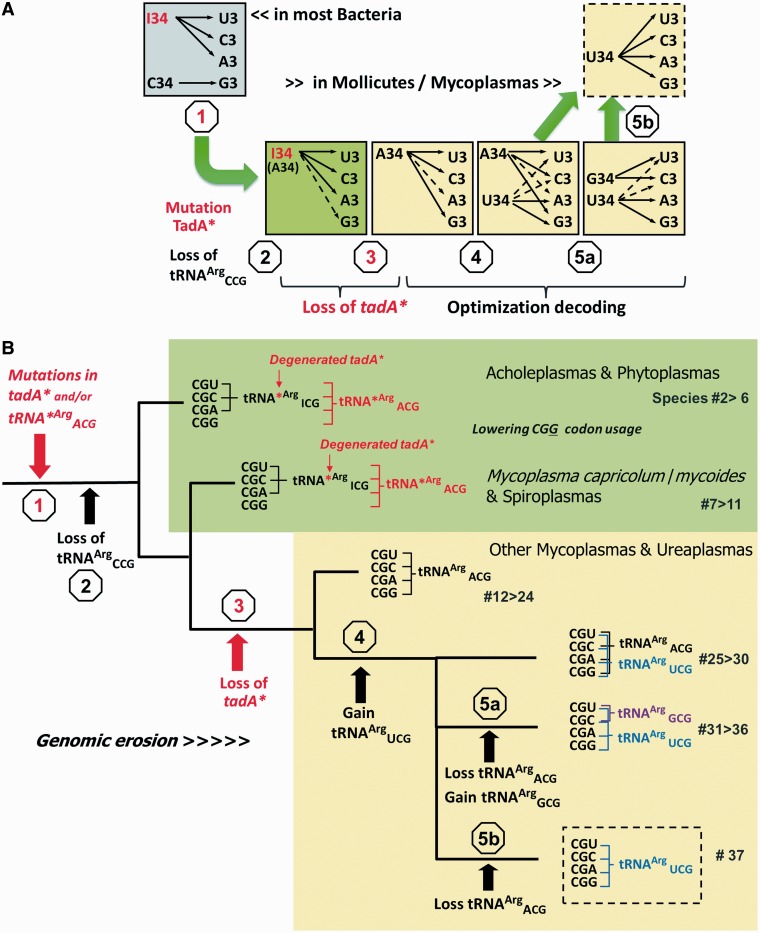
Hypothetical scenario for the evolution of the CGN decoding system for arginine in Mollicutes. (**A**) Schematic view of the five sequential events leading from a ‘classical bacterial’ arginine decoding strategy involving two tRNA^Arg^, one with a wobble inosine-34 and the other with a wobble C_34_, to another Arg decoding strategy involving only one tRNA^Arg^ with an unmodified wobble U_34_. In *M. capricolum*, this latter situation exists in many other quartet decoding boxes (Leu, Val, Ser, Pro, Ala and Gly), as well as in most mitochondria of eukarya. (**B**) The same events as in A, but depicted within the Mollicute evolutionary framework. Because of the degenerated TadA*, partial A-to-I deamination occurs at the first anticodon position of tRNA^Arg^_A__CG_ (Step 1), generating a situation where a mixture of both deaminated (in black) and non-deaminated tRNA^Arg^ (in red) molecules co-exist in the cell. In addition to the three synonymous arginine codons normally decoded by I_34_-containing tRNA^Arg^_I__CG_, tRNA^Arg^_A__CG_ also decodes the CGG codon, but probably inefficiently (see text). The gene encoding tRNA^Arg^_C__CG_ could then be lost (Step 2), along with the gene encoding tad* (Step 3). Further reorganization of the tRNA repertoire could occur by gaining an extra U_34_-containing tRNA^Arg^_U__CG_ (Step 4). The original A_34_-containing tRNA^Arg^_A__CG_ can undergo a mutation in its anticodon to generate a G_34_-containing tRNA^Arg^_G__CG_ (Step 5a), or simply be lost (Step 5b). The species of Mollicutes in which these different events occurred are indicated by numbers, corresponding to the organisms listed in Table 1. The phylogenetic relationships among the different Mollicutes were adapted from the literature (29–31).

In *M. capricolum*, only two kinds of tRNA^Arg^ exist for reading the six arginine codons (four in the quartet and two in the duet family boxes). One tRNA contains an anticodon with a wobble inosine (tRNA^Arg^_I__CG_) and the other contains an anticodon with a modified wobble uridine (cmnm^5^U_34_, tRNA^Arg^_U*__CU_) (41). Because of the wobble inosine-34, tRNA^Arg^_I__CG_ was expected to read only the three arginine codons ending with U, C or A of the quartet family box (8,9,16). Paradoxically, a tRNA^Arg^ harboring the anticodon CCG, needed to read the remaining fourth arginine codon CGG, as found in the majority of other bacteria (Table 1, items 1a–1j), was absent (41). Here, we demonstrated that a small fraction of the cellular A_34_-containing tRNA^Arg^_A__CG_ precursor is not enzymatically deaminated in *M. capricolum*. The key point of our report is the correlation with a few characteristic amino acid variants that exist within the active sites of the TadA’s of *M. capricolum* and other Mollicutes, as compared with other well characterized bacterial TadA’s considered as references. We hypothesize that these point mutations are needed for reducing the enzymatic activity of the tRNA:A_34_ deamination (degenerate TadA*), allowing the accumulation of a small but sufficient amount of the non-deaminated A_34_-containing tRNA^Arg^_A__CG_, which is competent for reading all four arginine codons of the quartet CGN decoding box (Step 1 in Figure 5A). To use a term that was first applied in the case of unmodified U_34_-containing tRNAs, this decoding strategy would correspond to a sort of ‘superwobbling’, facilitating the translation of synonymous codons with a reduced set of tRNAs (40). Therefore, the useless C_34_-containing tRNA^Arg^_C__CG_ can be lost (Step 2 in Figure 5A). This process was probably facilitated by limiting the usage of the problematic CGG codon (Step 2). Indeed, among 1163 CGN codons, only 6 such rare CGG codons, each in different mRNAs, were detected in the ORFs of *M. capricolum*.

### Reading arginine codons in other Mollicutes (Spiroplasma and Acholeplasma/Phytoplasma)

Combining our comparative genome analysis with information about the evolutionary origin of Mollicutes (29,30) revealed that the decoding strategy for *M. capricolum* is still in use in all Mollicutes of Groups I (Spiroplasmas, items 7–11) and IV (Acholeplasmas/Phytoplasmas, items 2–6), as shown in Table 1 and the green background in Figure 5B. Obviously, the two events (Steps 1 and 2 described earlier in the text) occurred early in evolution, almost at the root of the monophyletic Mollicute tree. These Mollicutes currently have the same original set of two genes: one gene encoding an A_34_-containing tRNA^Arg^_A__CG_ for reading a minimum number of CGN codons, and a second one harboring a U*CU anticodon (tRNA^Arg^_U*__CU_) for reading the other most frequently used arginine codons AGA and AGG; only the original TadA is now the mutant TadA*.

### Further stepwise evolution of the decoding strategy in Hominis and Pneumoniae

To become less dependent on the activity of the hypothetical degenerate TadA*, a subset of the newly evolved Mollicutes lost the degenerated tadA* gene (Step 3). This new evolutionary event occurred before the divergence into Groups III (Hominis) and II (Pneumoniae), items 12–37—all indicated with a yellow background in Figure 5A and B. Interestingly, the usage of the earlier problematic and rare CGG codon in these newly evolved Mollicutes became more frequent again, confirming that a Mollicute lacking the *tadA* gene and encoding an unmodified wobble A_34_-containing tRNA^Arg^_A__CG_ (items 12–24 in Figure 5) is perfectly viable because of its ability to read all four Arg-CGN codons.

In a subset (items 25–37) of Groups II and III (Hominis/Pneumoniae), the reading of the four synonymous arginine CGN codons was probably improved by gaining a new U_34_-containing tRNA^Arg^_U__CG_ (Step 4 in Figure 5A and B). This U_34_-containing tRNA^Arg^_U__CG_ could have originated in diverse manners. It may have arisen from the duplication of the gene encoding A_34_-containing tRNA^Arg^_A__CG_, followed by a few mutations, including the wobble A_34_-to-U_34_. It may also have resulted from duplication and subsequent recruitment/mutation of a gene encoding a tRNA possibly from the other duet Arg-AGR coding box, or belonging to another amino acid coding box. The mutations in the tRNA^Arg^_A__CG_ substrate itself may modulate the efficiency of A_34_-deamination and ultimately play a role in decoding all four arginine CGN codons (Supplementary Figure S2). Unfortunately, a phylogenetic analysis of all of the tRNA genes retrieved from the 36 Mollicutes examined did not allow us to confidently determine which one of these two alternatives prevailed because of the low-bootstrap values in constructing such phylogenetic trees with relatively short tRNAs, including many conserved and semi-conserved nucleotides and invariant regions under strong selective pressure (46,47).

Among the few species (items 25–30) of Groups II and III (Hominis/Pneumoniae), the four arginine CGN codons are read by a tRNA^Arg^ pair, one with a non-deaminated wobble A_34_ and the other with a wobble U_34_ (Figure 5, yellow background; U_34_ is probably not modified, see later in the text). This decoding strategy is also the one used presently for reading the four CGN codons as arginine in a few other non-Mollicute bacteria, such as *Clostridium perfringens*, *Chlamydia trachomatis*, *Geobacter metalloreducens* and *Haloplasma contractile*, the four CUN codons as leucine in *Lactococcus lactis*, and as mentioned in the ‘Introduction’ section, for reading the four ACN codons as threonine in *M. capricolum* (11,41).

Other species of Group II-Pneumoniae (items #31-36, including *M. pulmonis*) continued to evolve by using a slightly different decoding strategy (Step 5a). In these species, the CGN codons are now read by another type of tRNA^Arg^ set, one with a wobble U_34_ and the other one with G_34_ (Figure 5, yellow background). Because of the close sequence homology between the new G_34_-containing tRNA^Arg^ and the A_34_-containing tRNA^Arg^ in the other Pneumoniae (data not shown), this new G_34_-containing tRNA^Arg^ is believed to have arisen via a simple A_34_-to-G_34_ mutation and additional base mutations within the rest of the tRNA^Arg^_A__CG_ structure. This last decoding strategy is most frequently used in bacteria for decoding the sense codons of quartet synonymous codon boxes, at least in bacteria with moderate or low G + C content in their ORFs, as in *Borrelia burgdorferi, Campylobacter jejuni, Helicobacter pylori, Treponema palladium, Thermotoga maritima* and a few others (11).

Finally, one Mycoplasma in Group II, *M. haemofelis* (item 37 in Table 1), lost the ancient A_34_-(or G_34_)-containing tRNA^Arg^_A__CG_ (Step 5b); thus, it has only one U_34_-containing tRNA^Arg^_U__CG_ for reading the four synonymous Arg-CGN codons. This situation corresponds to the minimal set of tRNA^Arg^ that a Mollicute can use to continue decoding all CGN codons as arginine, with no need for the enzyme TadA and probably with better efficiency than that with a single A_34_-containing tRNA^Arg^_A__CG_. This decoding strategy was also used in other quartet decoding boxes corresponding to Leu, Val, Ser, Pro, Ala and Gly in *M. capricolum*, *M. mycoides* and the mitochondria of *S. cerevisiae* and mammals (24,39,41); reviewed in (5,48). The sequences of the corresponding tRNAs revealed the presence of an unmodified U_34_ in their anticodons (15).

### Analogy to a similar situation in the chloroplasts of higher plants

Gene knockout experiments in the plastids of the moss *Physcomitrella patens* demonstrated the dispensability of the C_34_-containing tRNA^Arg^_C__CG_, whereas the chloroplastic A_34_-containing tRNA^Arg^_A__CG_ and the chloroplastic TadA enzyme are encoded in the plastid and nuclear genomes, respectively (49). This situation corresponds to that of the Groups I (Spiroplasmas) and IV (Acholeplasmas/Phytoplasmas) Mollicutes (Table 1), which also lack C_34_-tRNA^Arg^_C__CG_ (see earlier in the text). On the other hand, the chloroplasts of *A. thaliana* lack C_34_-tRNA^Arg^_C__CG_, and only two kinds of tRNA^Arg^ are encoded on the plastid genome: one with the anticodon ACG and the other one with the anticodon UCU. In this species, the inhibition of the chloroplastic *tadA* gene expression by RNAi (not the cytoplasmic Tad2/Tad3) allows plant survival, and only the chloroplast translation and photosynthesis activities were hindered (17,18). This situation corresponds to the one described earlier in the text for the Mollicutes of Hominis Group III. By analogy with our results in the case of *M. capricolum*, we anticipate that in the chloroplasts of wild-type *A. thaliana*, and probably in other plant plastids, a fraction of the chloroplastic A_34_-containing tRNA^Arg^_A__CG_ also remains naturally unmodified, allowing superwobbling for decoding all CGN codons, including the rare Arg-CGG (16).

### Evolutionary scenario of the Mollicute decoding process

The scenario proposed in Figure 5 illustrates the evolvability of the decoding process. However, changing the decoding strategy during cellular evolution depends on a series of sequentially ordered events, such as point mutations in modification enzymes (probably also in the tRNA), gene loss, gene duplication and possibly the recruitment of a gene encoding a tRNA from another decoding box. The driving forces of this evolutionary process are almost certainly the efficacy and accuracy of translation. The sequence of events we have proposed, to explain the elimination of the essential deaminase TadA in Mollicutes, also applies to the essential tRNA–lysidine synthase TilS, responsible for the k^2^C modification at the wobble position 34 of tRNA^Ile^_C__AU_. Indeed, although it is encoded in the genomes of 35 Mollicutes, the *tilS* gene is notably absent in *Mycoplasma mobile*, with a concomitant change in the sequence of the minor tRNA^Ile^ that decodes AUA codons, from a CAU to a UAU anticodon (50,51). A similar cellular strategy has been experimentally verified in the case of *B. subtilis*, after the deletion of its essential *tilS* (52).

Finally, the idea of first reducing the activity of an enzyme (here, TadA or TilS) by point mutations, before its complete loss later in evolution, is reminiscent of recent work describing the progressive degeneration of aminoacyl-tRNA synthetases in *M. mobile* and other closely related Mycoplasmas of Group III-Hominis (53,54). In these cases, the degenerated aminoacyl-tRNA synthetases, while still performing the normal aminoacylation function, occasionally misacylate the cognate tRNA with a non-cognate amino acid. This allows the generation of a small number of cellular proteins with an incorrect amino acid substitution (statistical mutations). It was proposed that such misacylation reactions, if they are not too frequent, would provide an advantage to the Mycoplasma, which are indeed evolving faster than other extant bacteria by producing a more homogeneous proteome (55).

## SUPPLEMENTARY DATA

Supplementary Data are available at NAR Online: Supplementary Tables 1–2 and Supplementary Figures 1–3.

## FUNDING

Naito Foundation [2011-164 to Y.B.]; Daiichi-Sankyo Foundation of Life Science [12-039 to Y.B.]; X-ray Free Electron Laser Priority Strategy Program, from the Ministry of Education, Culture, Sports, Science and Technology (MEXT) of Japan (to Y.B.). H.G. holds the position of Emeritus Scientist at the CNRS in Gif-sur-Yvette, France, in the laboratory of Dominique Fourmy and Satoko Yoshizawa. Funding for open access charge: Naito Foundation [2011-164 to Y.B.].

*Conflict of interest statement.* None declared.

## Supplementary Material

Supplementary Data
