# Supplementary material for: Life without tRNAArg–adenosine deaminase TadA: evolutionary consequences of decoding the four CGN codons as arginine in Mycoplasmas and other Mollicutes
Source: Nucleic Acids Res. 2013 May 8;41(13):6531–43. doi: 10.1093/nar/gkt356 (PMC3711424; doi:10.1093/nar/gkt356)
Supplement: Supplementary Data [file supp_41_13_6531__index.html]

Life without tRNAArg–adenosine deaminase TadA: evolutionary consequences of decoding the four CGN codons as arginine in Mycoplasmas and other Mollicutes — Life without tRNAArg–adenosine deaminase TadA: evolutionary consequences of decoding the four CGN codons as arginine in Mycoplasmas and other Mollicutes — Supplementary Data 

# Life without tRNAArg–adenosine deaminase TadA: evolutionary consequences of decoding the four CGN codons as arginine in Mycoplasmas and other Mollicutes

## Supplementary Data

files

**Files in this Data Supplement:**

- Supplementary Data - pdf file
