# Supplementary material for: Life without tRNAArg–adenosine deaminase TadA: evolutionary consequences of decoding the four CGN codons as arginine in Mycoplasmas and other Mollicutes
Source: Nucleic Acids Res. 2013 May 8;41(13):6531–43. doi: 10.1093/nar/gkt356 (PMC3711424; doi:10.1093/nar/gkt356)
Supplement: Supplementary Data [file supp_gkt356_nar-00698-h-2013-File003.pdf]

Supplementary materials concerning:

Life without $tRNA^{Arg}$-adenosine deaminase TadA: evolutionary consequences of decoding the four CGN codons as arginine in Mycoplasmas and other Mollicutes

By: Shin-ichi Yokobori[1,*], Aya Kitamura[2], Henri Grosjean[3], and Yoshitaka Bessho[2,*]

[1]Laboratory of Extremophiles, Department of Applied Life Sciences, School of Life Sciences, Tokyo University of Pharmacy and Life Sciences, 1432-1 Horinouchi, Hachioji, Tokyo 192-0392, Japan

[2]RIKEN SPring-8 Center, Harima Institute, 1-1-1 Kouto, Sayo, Hyogo 679-5148, Japan

[3]Centre de Génétique Moléculaire, UPR 3404, CNRS, Associée à l'Université Paris-Sud 11, FRC 3115, 1 avenue de la Terrasse, 91190 Gif-sur-Yvette, France

*To whom correspondence should be addressed,

Shin-ichi Yokobori: yokobori@ls.toyaku.ac.jp

Tel: +81-42-676-5035, Fax: +81-42-676-7145

Yoshitaka Bessho: bessho@spring8.or.jp

Tel: +81-791-58-2891, Fax: +81-791-58-2892

List of Supplemental materials:

Supplementary Table ST1: All genomes analyzed and their references.

Supplementary Table ST2: List of protein genes using the CG<u>G</u> codon in Mollicutes of group I.

Legends of Supplementary Figures

Supplementary Figure SF1: Comparative sequence analysis of $tRNA^{Arg}$ genes for the CG<u>N</u> box from 10 bacteria and 36 Mollicutes.

Supplementary Figure SF2: Peculiarities of the $tRNA^{Arg}$ sequences from Mollicutes.

Supplementary Figure SF3: TadA within the anticodon stem of tRNA from *Staphylococcus* and some Hominis (III).

Supplementary Table ST1. All genomes analyzed and their references.

No.	*Species*	Host	Moll Group	Accession	Genome size	ORF#	G+C%	tDNA genes	Ref.
**1a**	*Escherichia coli* str. K-12 substr. MG1655	n.a	outer	NC_000913	4639675	4319	50.8	89	1
**1b**	*Nitrosomonas europaea* ATCC 19718	n.a	outer	NC_004757	2812094	2461	50.7	41	2
**1c**	*Aquifex aeolicus* VF5	n.a	outer	NC_000918	1551335	1529	43.5	44	3
**1d**	*Streptomyces avermitilis* MA 4680	n.a	outer	NC_003155	9025608	7580	70.7	68	4
**1e**	*Synechococcus elongatus* PCC 6301	n.a	outer	NC_006576	2696255	2523	55.5	45	5
**1f**	*Staphylococcus aureus* subsp. *aureus* Mu50	n.a	outer	NC_002758	2878529	2696	32.9	60	6
**1g**	*Bacillus cereus* ATCC 14579	n.a	outer	NC_004722	5411809	5234	35.3	108	7
**1h**	*Bacillus subtilis* subsp. *subtilis* str. 168	n.a	outer	NC_000964	4215606	4176	43.5	86	8
**1i**	*Listeria monocytogenes* EGDe	n.a	outer	NC_003210	2944528	2846	38.0	67	9
**1j**	*Oenococcus oeni* PSU1	n.a	outer	NC_008528	1780517	1691	37.9	43	10
2	*Acholeplasma laidlawii* PG-8A	Animals, soils	IV	NC_010163	1496992	1380	31.9	34	11
3	*Aster yellows witches'-broom phytoplasma* AYWB	Plants	IV	NC_007716	706569	671	26.9	31	12
4	*Candidatus Phytoplasma australiense*	Plants	IV	NC_010544	879959	684	27.4	35	13
5	*Candidatus Phytoplasma mali*	Plants	IV	NC_011047	601943	479	21.4	32	14
6	*Onion yellows phytoplasma* OY-M	Plants	IV	NC_005303	853092	750	27.8	31	15
7	*Mesoplasma florum* L1	Flower surface	I	NC_006055	793224	682	27.0	29	16
8	*M. capricolum* subsp. *capricolum* ATCC 27343	goat	I	NC_007633	1010023	812	23.8	30	17
9	*M. leachii* PG50	cattle	I	NC_014751	1008951	882	23.8	30	18
10	*M. mycoides subsp. mycoides* SC str. PG1	cattle	I	NC_005364	1211703	1017	24.0	30	19
11	*M. mycoides subsp. capri* LC str. 95010	goat	I	NC_015431	1153998	922	23.8	30	20
12	*M. agalactiae*	sheep	III	NC_006055	1006702	813	29.6	34	21
13	*M. agalactiae* PG2	sheep	III	NC_009497	877438	742	29.7	34	22
14	*M. arthritidis* 158L3-1	rat	III	NC_011025	820453	631	30.7	32	23
15	*M. bovis* PG45	cattle	III	NC_014760	1003404	765	29.3	34	24
16	*M. conjunctivae* HRC/581	caprine	III	NC_012806	846214	692	28.5	29	25
17	*M. crocodyli* MP145	crocodile	III	NC_014014	934379	689	27.0	34	26
18	*M. hominis* ATCC 23114	human	III	NC_013511	665445	523	27.1	33	27
19	*M. hyopneumoniae* 232	swine	III	NC_006360	892758	691	28.6	30	28
20	*M. hyopneumoniae* 7448	swine	III	NC_007332	920079	657	28.5	30	29
21	*M. hyopneumoniae* J	swine	III	NC_007295	897405	657	28.5	30	29
22	*M. hyorhinis* HUB-1	swine	III	NC_014448	839615	654	25.9	30	30
23	*M. mobile* 163K	fish	III	NC_006908	777079	633	25.0	28	31
24	*M. synoviae* 53	swine	III	NC_007294	799476	659	28.5	34	29
25	*M. fermentans* JER	human	III	NC_014552	977524	797	26.9	36	32
26	*M. fermentans* M64	human	III	NC_014921	1118751	1050	26.9	36	33
27	*M. penetrans* HF-2	human	II	NC_004432	1358633	1037	25.7	29	34
28	*Ureaplasma parvum serovar* 3 str. ATCC 27815	human	II	NC_010503	751679	609	25.5	30	35
29	*Ureaplasma parvum serovar* 3 str. ATCC 700970	human	II	NC_002162	751719	614	25.5	30	36
30	*Ureaplasma urealyticum serovar* 10 str. ATCC 33699	human	II	NC_011374	874478	646	25.8	30	37
31	*M. gallisepticum* str. R(low)	birds	II	NC_004829	1012800	763	31.5	32	38
32	*M. genitalium* G37	human	II	NC_000908	580076	475	31.7	36	39
33	*M. pneumoniae* M129	human	II	NC_000912	816394	689	40.0	37	40
34	*M. pulmonis* UAB CTIP	mouse	III	NC_002771	963879	782	26.6	29	41
35	*M. suis* KI3806	swine	II	NC_015153	709270	794	31.1	32	42
36	*M. suis* str. Illinois	swine	II	NC_015155	742431	844	31.1	32	43
37	*M. haemofelis* str. Langford 1	cat	II	NC_014970	1147259	1545	38.9	31	44

Accession numbers of each species, their subfamily and their host (in the cases of parasitic Mollicutes).

References for genomes.

1 Blattner FR, Plunkett G 3rd, Bloch CA, Perna NT, Burland V, Riley M, Collado-Vides J, Glasner JD, Rode CK, Mayhew GF, Gregor J, Davis NW, Kirkpatrick HA, Goeden MA, Rose DJ, Mau B, Shao Y (1997) The

complete genome sequence of *Escherichia coli* K-12. *Science* 277: 1453-1462.

2 Chain P, Lamerdin J, Larimer F, Regala W, Lao V, Land M, Hauser L, Hooper A, Klotz M, Norton J, Sayavedra-Soto L, Arciero D, Hommes N, Whittaker M, Arp D (2003) Complete genome sequence of the ammonia-oxidizing bacterium and obligate chemolithoautotroph *Nitrosomonas europaea*. *J Bacteriol* 185: 2759-2773.

3 Deckert G, Warren PV, Gaasterland T, Young WG, Lenox AL, Graham DE, Overbeek R, Snead MA, Keller M, Aujay M, Huber R, Feldman RA, Short JM, Olsen GJ, Swanson RV (1998) The complete genome of the hyperthermophilic bacterium *Aquifex aeolicus*. *Nature* 392: 353-358.

4 Ikeda H, Ishikawa J, Hanamoto A, Shinose M, Kikuchi H, Shiba T, Sakaki Y, Hattori M, Omura S. (2003) Complete genome sequence and comparative analysis of the industrial microorganism *Streptomyces avermitilis*. *Nat Biotechnol* 21:526-531.

5 Sugita C, Ogata K, Shikata M, Jikuya H, Takano J, Furumichi M, Kanehisa M, Omata T, Sugiura M, Sugita M (2007) Complete nucleotide sequence of the freshwater unicellular cyanobacterium *Synechococcus elongatus* PCC 6301 chromosome: gene content and organization. *Photosynth Res* 93: 55-67.

6 Kuroda M, Ohta T, Uchiyama I, Baba T, Yuzawa H, Kobayashi I, Cui L, Oguchi A, Aoki K, Nagai Y, Lian J, Ito T, Kanamori M, Matsumaru H, Maruyama A, Murakami H, Hosoyama A, Mizutani-Ui Y, Takahashi NK, Sawano T, Inoue R, Kaito C, Sekimizu K, Hirakawa H, Kuhara S, Goto S, Yabuzaki J, Kanehisa M, Yamashita A, Oshima K, Furuya K, Yoshino C, Shiba T, Hattori M, Ogasawara N, Hayashi H, Hiramatsu K (2001) Whole genome sequencing of methicillin-resistant *Staphylococcus aureus*. *Lancet* 357: 1225-1240.

7 Ivanova N, Sorokin A, Anderson I, Galleron N, Candelon B, Kapatral V, Bhattacharyya A, Reznik G, Mikhailova N, Lapidus A, Chu L, Mazur M, Goltsman E, Larsen N, D'Souza M, Walunas T, Grechkin Y, Pusch G, Haselkorn R, Fonstein M, Ehrlich SD, Overbeek R, Kyrpides N (2003) Genome sequence of *Bacillus cereus* and comparative analysis with *Bacillus anthracis*. *Nature* 423: 87-91.

8 Kunst F, Ogasawara N, Moszer I, Albertini AM, Alloni G, Azevedo V, Bertero MG, Bessieres P, Bolotin A, Borchert S, Borriss R, Boursier L, Brans A, Braun M, Brignell SC, Bron S, Brouillet S, Bruschi CV, Caldwell B, Capuano V, Carter NM, Choi SK, Codani JJ, Connerton IF, Cummings NJ, Daniel RA, Denizot F, Devine KM, Dusterhoft A, Ehrlich SD Emmerson PT, Entian KD, Errington J, Fabret C, Ferrari E, Foulger D, Fritz C, Fujita M, Fujita Y, Fuma S, Galizzi A, Galleron N, Ghim SY, Glaser P, Goffeau A, Golightly EJ, Grandi G, Guiseppi G, Guy BJ, Haga K, Haiech J, Harwood CR, Henaut A, Hilbert H, Holsappel S, Hosono S, Hullo MF, Itaya M, Jones L, Joris B, Karamata D, Kasahara Y, Klaerr-Blanchard M, Klein C, Kobayashi Y, Koetter P, Koningstein G, Krogh S, Kumano M, Kurita K, Lapidus A, Lardinois S, Lauber J, Lazarevic V, Lee SM, Levine A, Liu H, Masuda S, Mauel C, Medigue C, Medina N, Mellado RP, Mizuno M, Moestl D, Nakai S, Noback M, Noone D, O'Reilly M, Ogawa K, Ogiwara A, Oudega B, Park SH, Parro V, Pohl TM, Portetelle D, Porwollik S, Prescott AM, Presecan E, Pujic P, Purnelle B, Rapoport G, Rey M, Reynolds S, Rieger M, Rivolta C, Rocha E, Roche B, Rose M, Sadaie Y, Sato T, Scanlan E, Schleich S, Schroeter R, Scoffone F, Sekiguchi J, Sekowska A, Seror SJ, Serror P, Shin BS, Soldo B, Sorokin A, Tacconi E, Takagi T, Takahashi H, Takemaru K, Takeuchi M, Tamakoshi,A, Tanaka,T, Terpstra,P, Tognoni,A, Tosato,V, Uchiyama,S, Vandenbol,M, Vannier,F, Vassarotti,A, Viari A, Wambutt R, Wedler E, Wedler H, Weitzenegger T, Winters P, Wipat A, Yamamoto H, Yamane K, Yasumoto K, Yata K, Yoshida K, Yoshikawa HF, Zumstein E , Yoshikawa H, Danchin A (1997) The complete genome sequence of the Gram-positive bacterium *Bacillus subtilis*. *Nature* 390: 249-256.

9 Glaser P, Frangeul L, Buchrieser C, Rusniok C, Amend A, Baquero F, Berche P, Bloecker H, Brandt P, Chakraborty T, Charbit A, Chetouani F, Couvé E, de Daruvar A, Dehoux P, Domann E, Domínguez-Bernal G, Duchaud E, Durant L, Dussurget O, Entian KD, Fsihi H, García-del Portillo F, Garrido P, Gautier L, Goebel W, Gómez-López N, Hain T, Hauf J, Jackson D, Jones LM, Kaerst U, Kreft J, Kuhn M, Kunst F, Kurapkat G, Madueno E, Maitournam A, Vicente JM, Ng E, Nedjari H, Nordsiek G, Novella S, de Pablos B, Pérez-Diaz JC, Purcell R, Remmel B, Rose M, Schlueter T, Simoes N, Tierrez A, Vázquez-Boland JA, Voss H, Wehland J, Cossart P (2001) Comparative genomics of *Listeria* species. *Science* 294: 849-852.

10 Makarova K, Slesarev A, Wolf Y, Sorokin A, Mirkin B, Koonin E, Pavlov A, Pavlova N, Karamychev V, Polouchine N, Shakhova V, Grigoriev I, Lou Y, Rohksar D, Lucas S, Huang K, Goodstein DM, Hawkins T,

Plengvidhya V, Welker D, Hughes J, Goh Y, Benson A, Baldwin K, Lee JH, Díaz-Muñiz I, Dosti B, Smeianov V, Wechter W, Barabote R, Lorca G, Altermann E, Barrangou R, Ganesan B, Xie Y, Rawsthorne H, Tamir D, Parker C, Breidt F, Broadbent J, Hutkins R, O'Sullivan D, Steele J, Unlu G, Saier M, Klaenhammer T, Richardson P, Kozyavkin S, Weimer B, Mills D (2006) Comparative genomics of the lactic acid bacteria. *Proc Natl Acad Sci USA* 103: 15611-15616.

11 Lazarev VN, Levitskii SA, Basovskii YI, Chukin MM, Akopian TA, Vereshchagin VV, Kostrjukova ES, Kovaleva GY, Kazanov MD, Malko DB, Vitreschak AG, Sernova NV, Gelfand MS, Demina IA, Serebryakova MV, Galyamina MA, Vtyurin NN, Rogov SI, Alexeev DG, Ladygina VG, Govorun VM. (2011) Complete genome and proteome of *Acholeplasma laidlawii*. *J Bacteriol* 193: 4943-4953.

12 Bai X, Zhang J, Ewing A, Miller SA, Jancso Radek A, Shevchenko DV, Tsukerman K, Walunas T, Lapidus A, Campbell JW, Hogenhout SA. (2006) Living with genome instability: the adaptation of phytoplasmas to diverse environments of their insect and plant hosts. *J Bacteriol* 188: 3682-3696.

13 Tran-Nguyen LT, Kube M, Schneider B, Reinhardt R, Gibb KS (2008) Comparative genome analysis of "Candidatus *Phytoplasma australiense*" (subgroup tuf-Australia I; rp-A) and "Ca. *Phytoplasma asteris*" Strains OY-M and AY-WB. *J Bacteriol* 190: 3979-3991.

14 Kube M, Schneider B, Kuhl H, Dandekar T, Heitmann K, Migdoll AM, Reinhardt R, Seemüller E (2008) The linear chromosome of the plant-pathogenic mycoplasma 'Candidatus *Phytoplasma mali*'. *BMC Genomics* 9: 306.

15 Oshima K, Kakizawa S, Nishigawa H, Jung HY, Wei W, Suzuki S, Arashida R, Nakata D, Miyata S, Ugaki M, Namba S (2003) Reductive evolution suggested from the complete genome sequence of a plant-pathogenic phytoplasma. *Nat Genet* 36: 27-29.

16 Birren BW, Stange-Thomann N, Hafez N, DeCaprio D, Fisher S, Butler J, Elkins T, Kodira CD, Major J, Wang S, Nicol R, Nusbaum C (2004) Direct submission to genbank/EMBL/DDBJ (Accession No. AE017263).

17 Glass JI, Lartigue C, Pfannkoch C, Baden-Tillson H, Smith HO, Venter JC, Roske K, Wise KS, Calcutt MJ, Nelson WC, Nierman WC (2005) Direct submission to genbank/EMBL/DDBJ (Accession No. CP000123).

18 Wise K, Calcutt MJ, Foecking MF, Madupu R, DeBoy RT, Roske K, Martin TR, Hvinden ML, Durkin AS, Glass J, Methe BA (2010) Direct submission to genbank/EMBL/DDBJ (Accession No. CP002108).

19 Westberg J, Persson A, Holmberg A, Goesmann A, Lundeberg J, Johansson KE, Pettersson B, Uhlén M (2004) The genome sequence of *Mycoplasma mycoides* subsp. *mycoides* SC type strain PG1T, the causative agent of contagious bovine pleuropneumonia (CBPP). *Genome Res* 14:221-227.

20 Thiaucourt F, Manso-Silvan L, Salah W, Barbe V, Vacherie B, Jacob D, Breton M, Dupuy V, Lomenech AM, Blanchard A, Sirand-Pugnet P. (2011) *Mycoplasma mycoides*, from "mycoides Small Colony" to "capri". A microevolutionary perspective. *BMC Genomics* 12: 114.

21 Birren BW, Stange-Thomann N, Hafez N, DeCaprio D, Fisher S, Butler J, Elkins T, Kodira CD, Major J, Wang S, Nicol R and Nusbaum C (2004) Direct submission to genbank/EMBL/DDBJ (Accession No. AE017263).

22 Sirand-Pugnet P, Lartigue C, Marenda M, Jacob D, Barré A, Barbe V, Schenowitz C, Mangenot S, Couloux A, Segurens B, de Daruvar A, Blanchard A, Citti C (2007) Being pathogenic, plastic, and sexual while living with a nearly minimal bacterial genome. *PLoS Genet* 3: e75.

23 Dybvig K, Zuhua C, Lao P, Jordan DS, French CT, Tu AH, Loraine AE (2008) Genome of *Mycoplasma arthritidis*. *Infect Immun* 76: 4000-4008.

24 Wise KS, Calcutt MJ, Foecking MF, Röske K, Madupu R, Methé BA (2011) Complete genome sequence of *Mycoplasma bovis* type strain PG45 (ATCC 25523). *Infect Immun* 79: 982-983.

25 Calderon-Copete SP, Wigger G, Wunderlin C, Schmidheini T, Frey J, Quail MA, Falquet L (2009) The Mycoplasma conjunctivae genome sequencing, annotation and analysis. *BMC Bioinformatics* 10 Suppl 6: S7.

26 Brown DR, Farmerie WG, May M, Benders GA, Durkin AS, Hlavinka K, Hostetler J, Jackson J, Johnson J, Miller RH, Paralanov V, Radune D, Szczypinski B, Glass JI (2011) Genome sequences of *Mycoplasma alligatoris* A21JP2T and *Mycoplasma crocodyli* MP145T. *J Bacteriol* 193: 2892-2893.

27 Pereyre S, Sirand-Pugnet P, Beven L, Charron A, Renaudin H, Barré A, Avenaud P, Jacob D, Couloux A,

Barbe V, de Daruvar A, Blanchard A, Bébéar C (2009) Life on arginine for *Mycoplasma hominis*: clues from its minimal genome and comparison with other human urogenital mycoplasmas. *PLoS Genet* 5: e1000677.

28 Minion FC, Lefkowitz EJ, Madsen ML, Cleary BJ, Swartzell SM, Mahairas GG (2004) The genome sequence of *Mycoplasma hyopneumoniae* strain 232, the agent of swine mycoplasmosis. *J Bacteriol* 186: 7123-7133.

29 Vasconcelos AT, Ferreira HB, Bizarro CV, Bonatto SL, Carvalho MO, Pinto PM, Almeida DF, Almeida LG, Almeida R, Alves-Filho L, Assunção EN, Azevedo VA, Bogo MR, Brigido MM, Brocchi M, Burity HA, Camargo AA, Camargo SS, Carepo MS, Carraro DM, de Mattos Cascardo JC, Castro LA, Cavalcanti G, Chemale G, Collevatti RG, Cunha CW, Dallagiovanna B, Dambrós BP, Dellagostin OA, Falcão C, Fantinatti-Garboggini F, Felipe MS, Fiorentin L, Franco GR, Freitas NS, Frías D, Grangeiro TB, Grisard EC, Guimarães CT, Hungria M, Jardim SN, Krieger MA, Laurino JP, Lima LF, Lopes MI, Loreto EL, Madeira HM, Manfio GP, Maranhão AQ, Martinkovics CT, Medeiros SR, Moreira MA, Neiva M, Ramalho-Neto CE, Nicolás MF, Oliveira SC, Paixão RF, Pedrosa FO, Pena SD, Pereira M, Pereira-Ferrari L, Piffer I, Pinto LS, Potrich DP, Salim AC, Santos FR, Schmitt R, Schneider MP, Schrank A, Schrank IS, Schuck AF, Seuanez HN, Silva DW, Silva R, Silva SC, Soares CM, Souza KR, Souza RC, Staats CC, Steffens MB, Teixeira SM, Urmenyi TP, Vainstein MH, Zuccherato LW, Simpson AJ, Zaha A. (2005) Swine and poultry pathogens: the complete genome sequences of two strains of *Mycoplasma hyopneumoniae* and a strain of *Mycoplasma synoviae*. *J Bacteriol* 187(16):5568-5577.

30 Liu W, Fang L, Li S, Li Q, Zhou Z, Feng Z, Luo R, Shao G, Wang L, Chen H, Xiao S (2010) Complete genome sequence of *Mycoplasma hyorhinis* strain HUB-1. *J Bacteriol* 192: 5844-5845.

31 Jaffe JD, Stange-Thomann N, Smith C, DeCaprio D, Fisher S, Butler J, Calvo S, Elkins T, FitzGerald MG, Hafez N, Kodira CD, Major J, Wang S, Wilkinson J, Nicol R, Nusbaum C, Birren B, Berg HC, Church GM (2004) The complete genome and proteome of *Mycoplasma mobile*. *Genome Res* 14: 1447-1461.

32 Rechnitzer H, Brzuszkiewicz E, Strittmatter A, Liesegang H, Lysnyansky I, Daniel R, Gottschalk G, Rottem S (2011) Genomic features and insights into the biology of *Mycoplasma fermentans*. *Microbiology* 157 (Pt 3): 760-773.

33 Shu HW, Liu TT, Chan HI, Liu YM, Wu KM, Shu HY, Tsai SF, Hsiao KJ, Hu WS, Ng WV (2011) Genome sequence of the repetitive-sequence-rich *Mycoplasma fermentans* strain M64. *J Bacteriol* 193: 4302-4303

34 Sasaki Y, Ishikawa J, Yamashita A, Oshima K, Kenri T, Furuya K, Yoshino C, Horino A, Shiba T, Sasaki T, Hattori M (2004) The complete genomic sequence of *Mycoplasma penetrans*, an intracellular bacterial pathogen in humans. *Nucleic Acids Res* 30: 5293-5300.

35 Methe BA, Glass J, Waites K (2008) Genome sequence of *Ureaplasma parvum* serovar 3. Direct submission to genbank/EMBL/DDBJ (CP000942).

36 Glass JI, Lefkowitz EJ, Glass JS, Heiner CR, Chen EY, Cassell GH (2000) The complete sequence of the mucosal pathogen *Ureaplasma urealyticum*. *Nature* 407: 757-762.

37 Shrivastava S, Methe BA, Glass J, White K, Duffy LB (2008) Genome sequence of *Ureaplasma urealyticum* serovar 10 ATCC-33699. Direct submission to genbank/EMBL/DDBJ (CP001184).

38 Papazisi L, Gorton TS, Kutish G, Markham PF, Browning GF, Nguyen DK, Swartzell S, Madan A, Mahairas G, Geary SJ (2003) The complete genome sequence of the avian pathogen *Mycoplasma gallisepticum* strain R(low). *Microbiology* 149(Pt 9): 2307-2316.

39 Fraser CM, Gocayne JD, White O, Adams MD, Clayton RA, Fleischmann RD, Bult CJ, Kerlavage AR, Sutton G, Kelley JM, Fritchman RD, Weidman JF, Small KV, Sandusky M, Fuhrmann J, Nguyen D, Utterback TR, Saudek DM, Phillips CA, Merrick JM, Tomb JF, Dougherty BA, Bott KF, Hu PC, Lucier TS, Peterson SN, Smith HO, Hutchison CA 3rd, Venter JC (1995) The minimal gene complement of *Mycoplasma genitalium*. *Science* 270: 397-403.

40 Dandekar T, Huynen M, Regula JT, Ueberle B, Zimmermann CU, Andrade MA, Doerks T, Sánchez-Pulido L, Snel B, Suyama M, Yuan YP, Herrmann R, Bork P (2000) Re-annotating the *Mycoplasma pneumoniae* genome sequence: adding value, function and reading frames. *Nucleic Acids Res* 28: 3278-3288.

41 Chambaud I, Heilig R, Ferris S, Barbe V, Samson D, Galisson F, Moszer I, Dybvig K, Wróblewski H, Viari A, Rocha EP, Blanchard A (2001) The complete genome sequence of the murine respiratory pathogen

Mycoplasma pulmonis. *Nucleic Acids Res* 29: 2145-2153.

42 Oehlerking J, Kube M, Felder KM, Matter D, Wittenbrink MM, Schwarzenbach S, Kramer MM, Hoelzle K, Hoelzle LE (2011) Complete genome sequence of the hemotrophic *Mycoplasma suis* strain KI3806. *J Bacteriol* 193: 2369-2370.

43 Messick JB, Santos AP, Guimaraes AM (2011) Complete genome sequences of two hemotropic mycoplasmas, *Mycoplasma haemofelis* strain Ohio2 and *Mycoplasma suis* Strain Illinois. *J Bacteriol* 193: 2068-2069.

44 Barker EN, Helps CR, Peters IR, Darby AC, Radford AD, Tasker S (2011) Complete genome sequence of *Mycoplasma haemofelis*, a hemotropic mycoplasma. *J Bacteriol* 193: 2060-2061.

Supplementary Table ST2. List of protein genes using the CGG codon in Mollicutes of group I.

Species	Accession No.	Name of protein or gene	Location on the genome	Ordinal No. of codon where CGG appears
Mycoplasma capricolum subsp. *capricolum* ATCC 27343	YP_424030.1	mannitol-1-phosphate 5-dehydrogenase	35825-36829	265th
	YP_424045.1	adenine-specific DNA methylase	61692-62708	196th
	YP_424046.1	**dam (DNA adenine methylase)**	62638-63615	2nd
	YP_424226.1	**thiI (thiamine biosynthesis/tRNA modification protein) ThiI (s^{4}U8)**	288478-289665	28th
	YP_424498.1	lipoprotein	636367-635564	111th
	YP_424629.1	**truA (ψ39) (tRNA pseudouridine synthase A)**	795900-795151	126th
Mycoplasma mycoides subsp. *mycoides* SC str. PG1	***NP_975060.1***	***tadA (tRNA adenosine deaminase)***	***59846-59379***	***2nd***
	NP_975168.1	hypothetical protein	193136-193753	104th
	NP_975278.1	hypothetical protein	317821-319629	418th
	NP_975445.1	abc (ABC transporter, ATP-binding and permease protein)	521270-522544	191st
	NP_975473.1	pstB (phosphate ABC transporter ATP-binding protein)	554208-554906	33rd
	NP_975572.1	**mraW (S-adenosyl-methyltransferase)**	665013-664087	296th
	NP_975573.1	cell division protein MraZ	665423-665022	111th
	NP_975692.1	**truA (tRNA pseudouridine synthase A)**	823366-822611	126th
	NP_975831.1	hypothetical protein	981111-982085	323rd
	NP_975844.1	hypothetical protein	996291-997265	323rd
Mycoplasma mycoides subsp. *capri* LC str. 95010	YP_004399916.1	relaxase	271756-273000	91st
	YP_004400013.1	relaxase	409473-408229	91st
	YP_004400125.1	deoxynucleoside kinase	532154-532792	101st
	YP_004400147.1	ATP binding and permease	562002-563786	156th
	YP_004400173.1	**pstB (phosphate ABC transporter ATP binding protein)**	596042-596851	70th
	YP_004400264.1	**mraW (S-adenosyl-methyltransferase)**	697299-696373	296th
	YP_004400265.1	hypothetical protein	697709-697308	111th
	YP_004400372.1	**truA (tRNA pseudouridine synthase A)**	853032-852277	126th
	YP_004400519.1	hypothetical protein	1011785-1010610	48th
Mycoplasma leachii PG50	YP_004046785.1	GnsA/GnsB family protein	26834-29986	780th
	YP_004047046.1	**thiI (thiamine biosynthesis/tRNA modification protein)**	336643-337830	18th
	YP_004047307.1	**rimM (16S rRNA processing protein)**	657001-656513	94th

	YP_004047457.1	hypothetical protein CDS	802551-802787	54th
	YP_004047561.1	hypothetical protein CDS	915608-915045	176th
Mesoplasma florum L1	YP_053440.1	engA (GTP-binding protein)	225425-226732	233rd
	YP_053681.1	heat shock protein chaperone	516135-515233	31st

All open reading frames of the *Mycoplasma capricolum* subsp. *capricolum* ATCC 27343 (NC_007633), *M. mycoides* subsp. *mycoides* SC str. PG1 (NC_005364), *M. mycoides* subsp. *capri* LC str. 95010 (NC_015431), *M. leachii* PG50 (NC_014751), and *Mesoplasma florum* L1 (NC_006055) genomes were checked for the presence of CGG codons. All of the protein genes using CGG codons in these species are listed with their accession numbers. The names of the genes, their positions on the encoding genome, and the positions of CGG codon appearance (as the ordinal number of codons) were obtained from the public databases. The names of the genes encoding proteins involved in post-replication, post-transcription, and post-translation processes are indicated in bold. NP_975060.1 from *M. mycoides* subsp. *mycoides* SC str. PG1 has been corrected as "tadA (tRNA adenosine deaminase)" in bold and italics, from the original description of "codA (cytosine deaminase)" in the database. The presence of a rare codon in mRNA often interrupts or at least slows translation, and may be part of a translation regulation system.

Legends of Supplementary Figures.

Supplementary Figure SF1. Comparative sequence analysis of $tRNA^{Arg}$ genes for the $CG\underline{N}$ box from 10 bacteria and 36 Mollicutes. The series of genes corresponding to $tRNA^{Arg}{}_{\underline{A}CG}$, in which the wobble A_{34} in the anticodon is most probably post-transcriptionally deaminated to inosine (I_{34}) by the enzyme TadA, are highlighted in the red box. Those corresponding to the $tRNA^{Arg}{}_{\underline{A}CG}$ genes, for which the wobble A_{34} is certainly not post-transcriptionally deaminated to I_{34}, because of the absence of the TadA enzyme, are highlighted in the blue box. In all other $tRNA^{Arg}$ sequences, C_{34}, G_{34} or T_{34} is present at the wobble position in the tDNA gene. The 3 bases of the anticodon sequences are also boxed in purple and indicated by AC. The numbers on the left side of the table correspond to the species names in Table 1. At the top of the alignment, the various parts of the tRNA molecule are indicated.

Supplementary Figure SF2. Peculiarities of the $tRNA^{Arg}$ sequences from Mollicutes. The various $tRNA^{Arg}$ genes, listed in Table 1 and Supplementary Figure SF1, are classified according to the identity of their wobble nucleotide-34 (A, U or G) and the type of Mollicute (from group I to group IV). The total number of sequences in each subgroup is indicated. Sequences were obtained from the tRNAdb database (1). Each sequence (from the 5'-end to the 3'-end) corresponds to only a portion of the unmodified tRNA transcript; e.g., the anticodon stem-loop, the dihydrouridine branch and the last nucleotide-73, preceding the CCA end of the tRNA cloverleaf. In the 3D-L-shaped structure of tRNA, G_{10} interacts with G_{45}, G_{26} interacts with nucleotide-44, and nucleotide-32 interacts with nucleotide-38 (indicated by dashed lines). The conserved G_{30}-C_{40} base pair plays the role of a pivot, to maintain a certain degree of rigidity in that particular region of the tRNA. In some cases where 2 to 4 nucleotides are mentioned at a given position, those depicted by smaller characters are less frequently encountered (for details see Supplementary Figure SF1). The nucleotides within the D-loop are not indicated and are replaced by a circle, except for A at position 20. Together, the purine-73

and C_{35} of the anticodon (indicated in red) and the A at position 20 constitute the major identity elements for tRNA^{Arg} recognition by the bacterial Arg-tRNA synthetase. The identities of the modified nucleotides in the anticodon loop and the proximal stem of the mature, fully modified tRNA^{Arg} are only known in the cases of *M. capricolum* (group I) and *A. laidlawii* (group IV). They are indicated in blue, with the acronyms of the corresponding tRNA modification enzymes catalyzing the reactions. Together with the wobble base-34, the base pairs or bases opposite positions 32-38, 27-43 and 11-24 (boxed in the Figure) were demonstrated to play an important role in the efficiency and accuracy of the decoding capability of tRNA^{Arg}. The nucleotides in the anticodon loop and the proximal stem (up to base pair 31-39) probably play a critical role in the efficiency of the deamination reaction catalyzed by the tRNA-A_{34} deaminase TadA.

Supplementary Figure SF3. TadA within the anticodon stem of tRNA from *Staphylococcus aureus* and some Hominis (III). A fragment of the tRNA:A_{34} deaminase TadA of *S. aureus* interacting with the anticodon stem-loops of *S. aureus* is shown, and it could represent any anticodon branch of tRNA^{Arg} belonging to the Mollicute groups I (Spiroplasma) and IV (Phytoplasma), but not group III (Hominis) (see Supplementary Figure SF2). This figure depicts the interaction of the part of the enzyme that contacts the base pairs 32-38 and 31-39 in the anticodon branch of the tRNA substrate. The subunits of the *S. aureus* TadA dimer are colored green and pink in the ribbon model. Conserved amino acids involved in the 32-38 tRNA interactions, as well as the base pairs 32-38 and 31-39 of tRNA, are shown in stick models. Other residues are shown in line models. The amino acid sequences are colored blue for *S. aureus*, and green for *M. capricolum*. The nucleotide sequences for the 31-39 and 32-38 pairs are brown for *S. aureus* and *M. capricolum*, and grey in parentheses for most Hominis (III) (Supplementary Figure SF2). Hydrogen bond distances are indicated between the amino acid side chains and the bases of the C_{32} and A_{38} nucleotides.

REFERENCE for Supplementary Figures

1. Jühling, F., Mörl, M., Hartmann, R.K., Sprinzl, M., Stadler, P.F. and Pütz, J. (2009) tRNAdb 2009: compilation of tRNA sequences and tRNA genes. *Nucleic Acids Res*, 37, D159-162.

Figure SC1

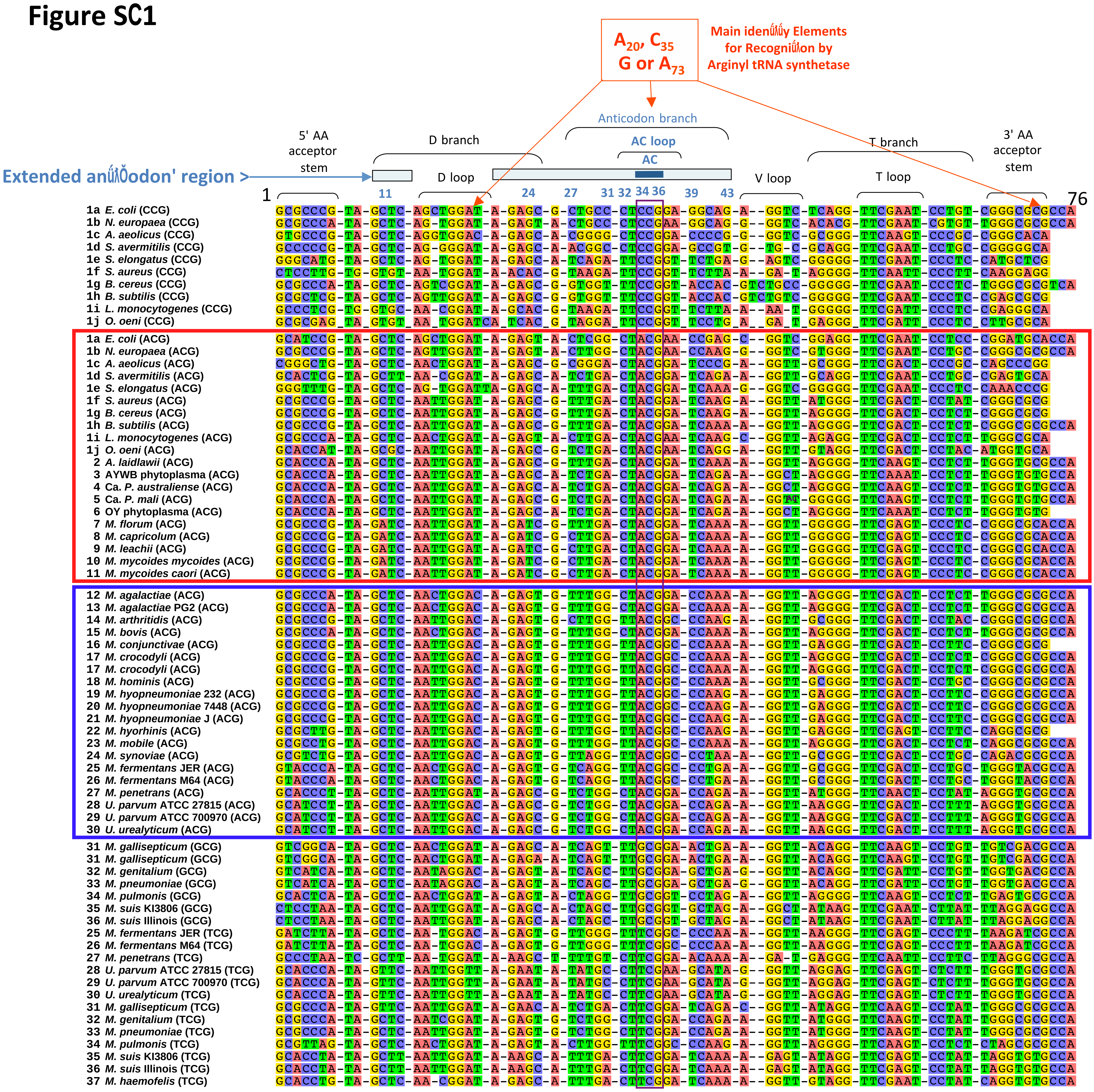

A20, C35
G or A73
Main identity Elements for Recognition by Arginyl tRNA synthetase
Anticodon branch
AC loop
AC
5' AA acceptor stem
D branch
D loop
T branch
T loop
3' AA acceptor stem
V loop
Extended anticodon' region
1
11
24
27
31 32 34 36
39
43
76
1a E. coli (CCG)
1b N. europaea (CCG)
1c A. aeolicus (CCG)
1d S. avermitilis (CCG)
1e S. elongatus (CCG)
1f S. aureus (CCG)
1g B. cereus (CCG)
1h B. subtilis (CCG)
1i L. monocytogenes (CCG)
1j O. oeni (CCG)
1a E. coli (ACG)
1b N. europaea (ACG)
1c A. aeolicus (ACG)
1d S. avermitilis (ACG)
1e S. elongatus (ACG)
1f S. aureus (ACG)
1g B. cereus (ACG)
1h B. subtilis (ACG)
1i L. monocytogenes (ACG)
1j O. oeni (ACG)
2 A. laidlawii (ACG)
3 AYWB phytoplasma (ACG)
4 Ca. P. australiense (ACG)
5 Ca. P. mali (ACG)
6 OY phytoplasma (ACG)
7 M. florum (ACG)
8 M. capricolum (ACG)
9 M. leachii (ACG)
10 M. mycoides mycoides (ACG)
11 M. mycoides caori (ACG)
12 M. agalactiae (ACG)
13 M. agalactiae PG2 (ACG)
14 M. arthritidis (ACG)
15 M. bovis (ACG)
16 M. conjunctivae (ACG)
17 M. crocodyli (ACG)
17 M. crocodyli (ACG)
18 M. hominis (ACG)
19 M. hyopneumoniae 232 (ACG)
20 M. hyopneumoniae 7448 (ACG)
21 M. hyopneumoniae J (ACG)
22 M. hyorhinis (ACG)
23 M. mobile (ACG)
24 M. synoviae (ACG)
25 M. fermentans JER (ACG)
26 M. fermentans M64 (ACG)
27 M. penetrans (ACG)
28 U. parvum ATCC 27815 (ACG)
29 U. parvum ATCC 700970 (ACG)
30 U. urealyticum (ACG)
31 M. gallisepticum (GCG)
31 M. gallisepticum (GCG)
32 M. genitalium (GCG)
33 M. pneumoniae (GCG)
34 M. pulmonis (GCG)
35 M. suis KI3806 (GCG)
36 M. suis Illinois (GCG)
25 M. fermentans JER (TCG)
26 M. fermentans M64 (TCG)
27 M. penetrans (TCG)
28 U. parvum ATCC 27815 (TCG)
29 U. parvum ATCC 700970 (TCG)
30 U. urealyticum (TCG)
31 M. gallisepticum (TCG)
32 M. genitalium (TCG)
33 M. pneumoniae (TCG)
34 M. pulmonis (TCG)
35 M. suis KI3806 (TCG)
36 M. suis Illinois (TCG)
37 M. haemofelis (TCG)

Figure SF2

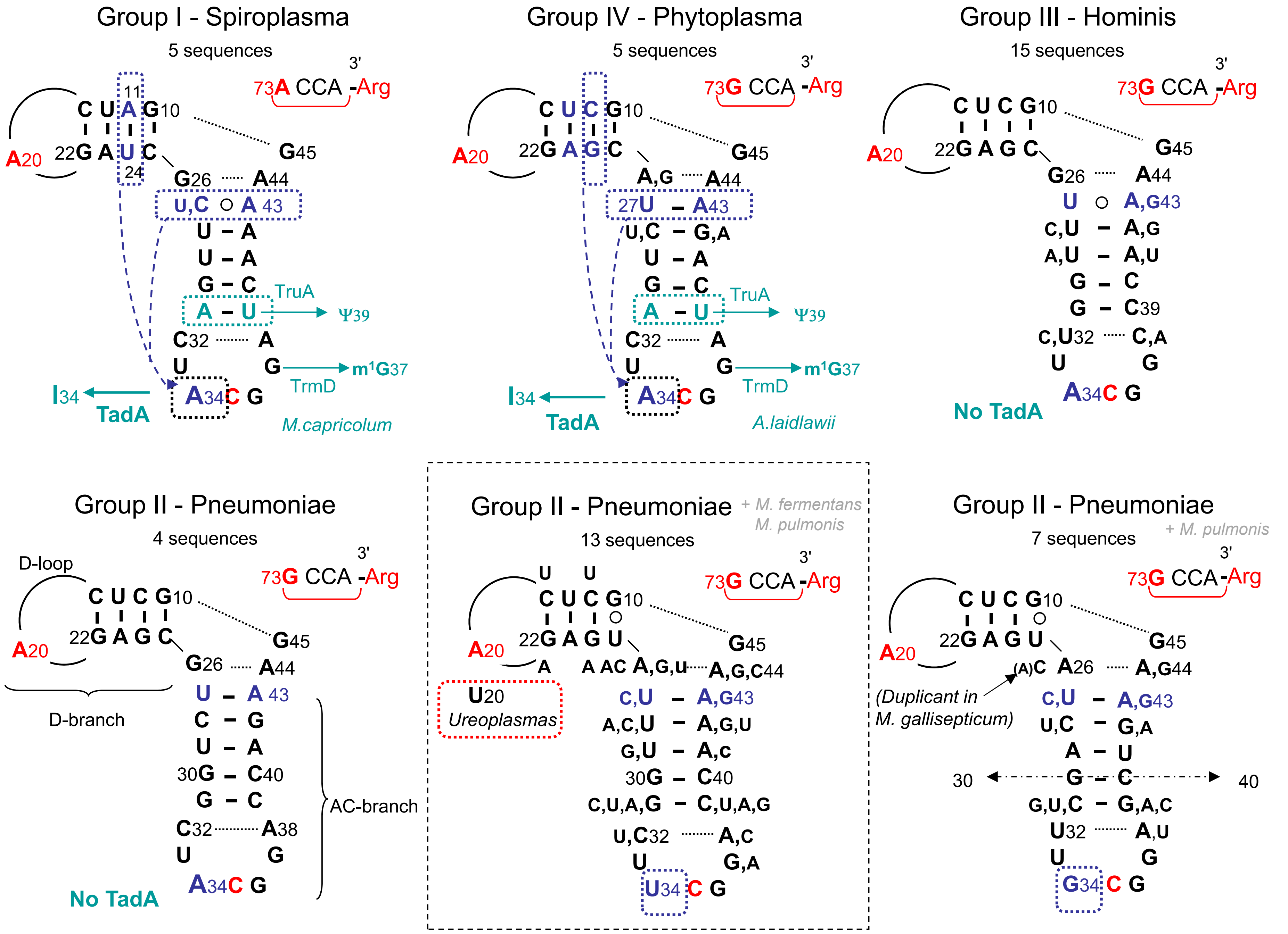

Group I - Spiroplasma
5 sequences
73A CCA -Arg
3'
A20
TruA
Ψ39
m1G37
TrmD
I34
TadA
M.capricolum
Group IV - Phytoplasma
5 sequences
73G CCA -Arg
TadA
A.laidlawii
Group III - Hominis
15 sequences
No TadA
Group II - Pneumoniae
4 sequences
D-loop
D-branch
AC-branch
No TadA
Group II - Pneumoniae
+ M. fermentans
M. pulmonis
13 sequences
U20
Ureoplasmas
Group II - Pneumoniae
+ M. pulmonis
7 sequences
(Duplicant in
M. gallisepticum)

Figure SF3

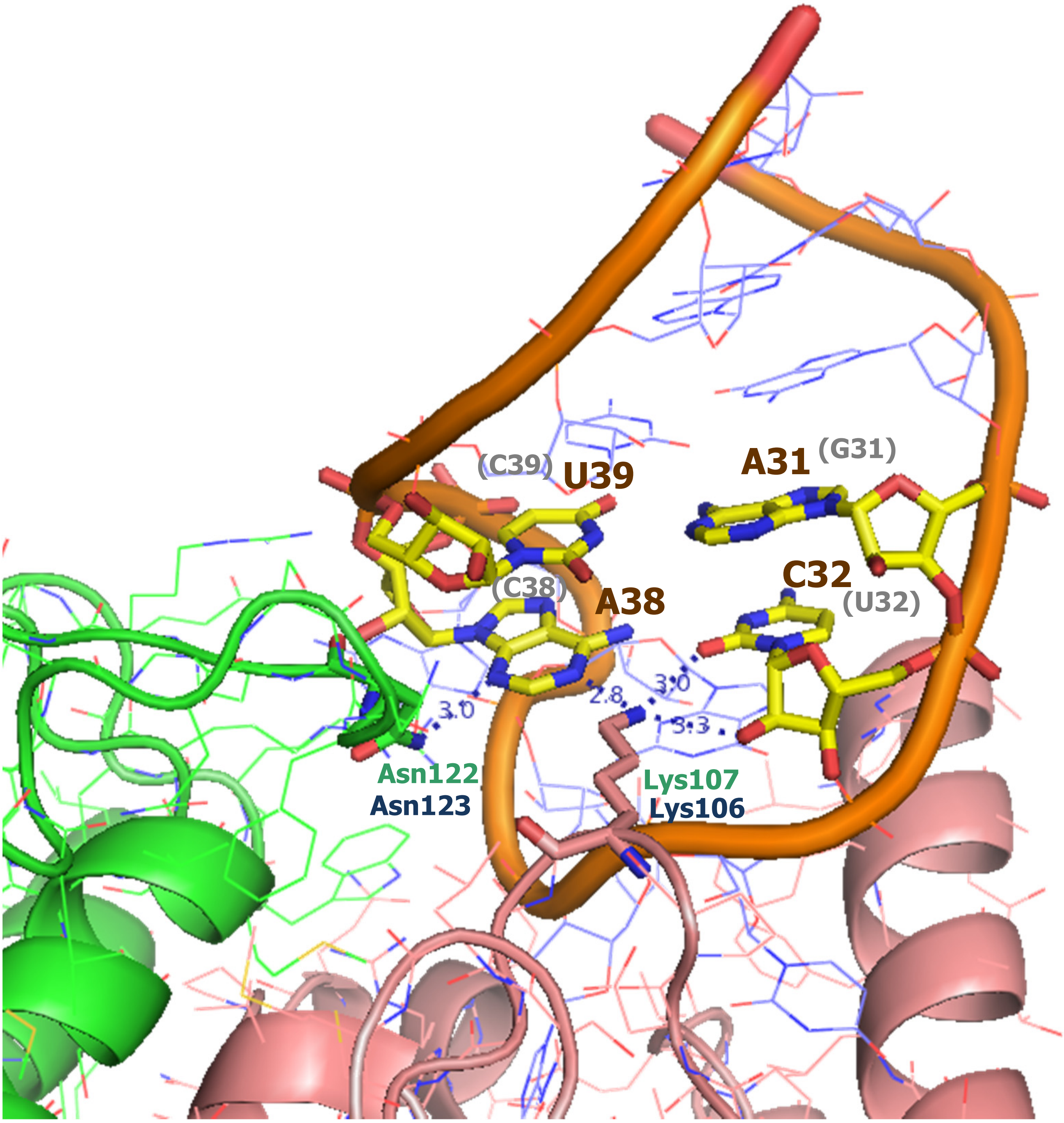
(C39)
U39
A31
(G31)
(C38)
A38
C32
(U32)
3.0
2.8
3.0
3.3
Asn122
Asn123
Lys107
Lys106
